# AI-driven personalization and impulsive buying in e-commerce: a bibliometric analysis of research trends among Millennials and Generation Z

**DOI:** 10.3389/frma.2026.1860513

**Published:** 2026-05-29

**Authors:** Alain Monica George, Rupa R, Shinta Sebastian, Vimal George Kurian, Jithin Joy

**Affiliations:** 1 Research and PG Department of Commerce, Marian College Kuttikkanam Autonomous, Kuttikkanam, India; 2 Department of Commerce, CMS College, Kottayam, India

**Keywords:** AI-driven personalization, artificial intelligence, bibliometric analysis, e-commerce, Generation Z, impulsive buying behavior, Millennials

## Abstract

**Objectives:**

The research is intended to chart the research space of artificial intelligence-based personalization and impulsive purchasing behavior in e-commerce, with a particular emphasis on Millennials and Generation Z. It aims to determine the trends of publications, the main themes, the sources of influence, the patterns of collaboration, as well as the new directions of the research.

**Methods:**

650 articles of Web of Science and 48 articles of Scopus were analyzed by Biblioshiny. Performance analysis, co-occurrence network analysis, thematic mapping, and source and authorship analysis were included in the analysis.

**Findings:**

The results indicate a definite increase in the number of publications over the past few years, which indicates growing interest among scholars in the AI and consumer behavior. In both data sets, the theme of artificial intelligence, technology adoption, and consumer behavior is predominant in the field, with trust, behavioral intention, and adoption proving to be key supporting concepts. The analysis of co-occurrence revealed the significant clusters with the focus on AI and technological improvement, consumer behavior, and engagement. Nonetheless, the direct correlation between AI-driven personalization and impulsive buying behavior is underrepresented, and the focus on Millennials and Generation Z has not been explicitly addressed yet.

**Conclusion:**

The study concludes that research in this area is currently increasing at an extremely fast rate, however, it remains in pieces and is still lacking a fully integrated conceptual framework. Though the literature covers AI and its impact on consumer behavior in a comprehensive manner, the behavioral consequence of impulsive buying by younger generations has been poorly covered in the literature.

**Implications:**

The results indicate that future research should address more directly how AI personalization processes relate to impulsive buying behaviors and should also investigate how generational differences influence the results. The research also provides valuable information to the researchers and practitioners with interest in the role of AI in digital commerce and consumer decision-making.

## 1 Introduction

The increased pace of artificial intelligence has drastically transformed online commerce by enabling more enhanced and personal experience to be given to the consumer (Avinash et al., 2025). The AI technologies enter online space to analyze large volumes of information about consumers and offer them individual recommendations, personalized advertising, and reactive digital experiences (Ghule, 2025). Such technological opportunities allow businesses to predict customer preferences and behavior more precisely, and therefore, raise the subject of customer interaction and influence their purchases in the virtual world (Ramesh and Murugan, 2025). Due to the emergence of recommendation engines, predictive analytics, and automated decision-making tools, the AI-based personalization engine of e-commerce has become an essential system in the increasingly popular e-commerce ecosystem (Rhayti, 2025; Patil and Patil, 2025). One among the most pressing research in the business and management literature is learning about artificial intelligence and its influence on consumer behavior as the digital platforms continue to advance (Hariharan et al., 2024). One of the behavioral outcomes of personalized digital environments is impulsive buying behavior (Pal, 2025). Impulse buying may be termed as spontaneous and unplanned buying in which one makes purchases without giving the subject much thinking (Singh and Gujar, 2025). The AI-based recommendation mechanism in the context of online shopping may result in impulse purchasing behavior; with its uncharacteristically topical products, individual deals, and time-related purchase cues, one will act (Fadilah et al., 2025). The algorithms of machine learning on the digital platforms will be used to interpret the past purchases, browsing history and current behavioral indicators to generate custom product suggestions (Fale, 2025). Such personalized contacts may increase the comfort and enjoyment of the consumers and create a circumstance that facilitates impulse purchase behavior (Hayrapetyan and Darbinyan, 2025). Thus, the interaction between the technologies of artificial intelligence and the impulsive consumer behavior have become topics of growing academic research in recent years (Shyni et al., 2025). However, as AI-driven personalization becomes deeply embedded in everyday online shopping journeys, there is a growing need to understand not only how these systems enhance convenience, but also how they may quietly reshape consumers' decision processes and impulse tendencies in digital marketplaces (Anwar et al., 2024; Nasution et al., 2025).

The impact of AI-based personalization on consumer behavior is especially significant to younger consumer groups like Millennials and Generation Z (Jit et al., 2025). These generational groups can be regarded as digitally native buyers that have been growing up in the technologically modernized surroundings and are very familiar with online shopping systems and online services (Radyi et al., 2024). Millennials and Generation Z are very engaged in social media, mobile applications as well as e-commerce sites, thus they are highly sensitive to customized digital marketing (Chen and Lasi, 2025). They are usually predisposed to the purchasing behavior through their acquaintance with algorithmic recommendations and interactive online interfaces, and so they are more likely to make impulsive purchases in online settings (Amin, 2025). As a result, scholars have been investigating the effects of artificial intelligence technologies on the decision-making processes of these generations of people more often (Cavazos-Arroyo and Máynez-Guaderrama, 2022).

Although there is an increasing amount of literature on the topic of artificial intelligence in e-commerce and online consumer behavior, the available studies are fragmented, in various disciplines and topics (Avinash et al., 2025). Research has investigated some of the facets of the use of AI in personalization, such as recommendation systems, consumer trust, user acceptance, and digital marketing strategies (Effendi et al., 2025). Nonetheless, a paucity of knowledge exists about the ways in which these streams of research are going to lead to the creation of knowledge on impulsive buying behavior among the consumers of Millennials and Generation Z (Hayrapetyan and Darbinyan, 2025). This gap is particularly important because Millennials and Generation Z represent the core of the global digital consumer base, and their purchasing decisions increasingly drive the growth, competitiveness, and sustainability of e-commerce markets (Hussain et al., 2025). Clarifying how AI-based personalization relates to their impulsive buying behavior can therefore inform more responsible platform design, guide ethical marketing practices, and support policy debates on data-driven consumer influence (Ahmed et al., 2026a; Wang et al., 2025). Moreover, the extreme growth of the publications in this field makes it difficult that the researchers should be able to determine the influential themes, the major contributors, and the new trends of research in a systematic way (Gunawan, 2025). Against this background, the present study is novel in two main ways. First, it focuses explicitly on the intersection of AI-driven personalization and impulsive buying behavior among Millennials and Generation Z, a link that existing work has only touched upon indirectly. Second, it uses a comprehensive bibliometric approach that combines Web of Science and Scopus data to systematically map this emerging field, rather than relying on narrative reviews or single-database searches that risk overlooking important patterns in themes, sources, and collaborations. Accordingly, there is a clear need for a structured overview that consolidates fragmented evidence, highlights how AI-personalization research connects to impulsive buying among younger consumers, and points to promising directions for future inquiry.

Bibliometric analysis in this sense presents a useful methodological strategy of mapping the intellectual organization and development of a subject of research in a systematic manner (Gunawan, 2025). Bibliometric methods permit the quantitative analysis of the scientific literature, which helps the researchers to study the trends in the productivity of the research and development of the topic, as well as collaboration networks in a particular field (Gupta, 2025; Soltani et al., 2023). Using bibliometric techniques to the literature on the topic of artificial intelligence-driven personalization and impulsive buying behavior, one can trace the most prevalent themes of the research, the journals that impact the overall topic and the emerging research horizons that dominate the field (Effendi et al., 2025). Thus, the current research paper represents a bibliometric analysis of the studies devoted to the exploration of the problem of artificial intelligence-driven personalization and impulsive buying behavior in the Millennials and Generation Z consumers. With the bibliographic data obtained with the help of the Web of Science and the help of Biblioshiny, the research investigates the literary output, the development of the topic, and the organization of the ideas of the literature published in 2020–2025.

The specific objectives of the study are as follows:

1. To analyze the growth, productivity, and key publication sources in the research domain.
2. To identify and map the core themes and conceptual structure of the field using bibliometric techniques.
3. To examine the evolution of research trends and collaboration patterns, with a focus on Millennials and Generation Z consumers.

The remainder of this paper is structured as follows: the next section outlines the bibliometric methods and data sources; this is followed by the presentation of results on publication trends, thematic structures, and collaboration patterns; the subsequent section discusses theoretical and practical implications, and the final section summarizes key conclusions and directions for future research.

## 2 Methods

### 2.1 Study design

The bibliometric research design was adopted in the present research to map the intellectual structure, the development of publication, and the change of the theme of personalization based on AI and impulsive buying behavior among Millennials and Generation Z. A bibliometric research design is appropriate in research studies that seek to examine the large volumes of literature due to its ability to quantify publication trends, collaboration patterns, source concentration, and conceptual relationships within a research field (Kuznetsov, 2023). This study employed bibliometric methods to establish how the topic has evolved over the years and how the literature relates artificial intelligence, consumer behavior, and digital commerce.

### 2.2 Data sources and search strategy

Web of Science was previously the main database in the study due to its wide coverage of citation and applicability in bibliometric studies. An inclusive keyword string was created to access the literature focusing on artificial intelligence, AI-based personalization, recommendation systems, impulse buying, internet consumer behavior, e-commerce, Millennials, and the Generation Z. The search was made in the title, abstract and key word areas to ensure that it was broad yet relevant to the subject. Enhance the validation of the study, the identical keyword string was utilized in Scopus, OpenAlex, and Dimensions as well. Scopus was stored as a secondary database to compare, and OpenAlex and Dimensions were utilized as additional coverage checks. The other searches in OpenAlex and Dimensions did not find any more articles, only the same article that was already available in the Scopus set, and it is unlikely that much more is covered by such a small, narrowly defined topic. Based on the result, Web of Science was kept as the primary corpus to consider in the general bibliometric mapping, whereas Scopus served as a secondary source that would help to check whether the primary patterns were shared by databases.

### 2.3 Study selection

**Figure 1:**
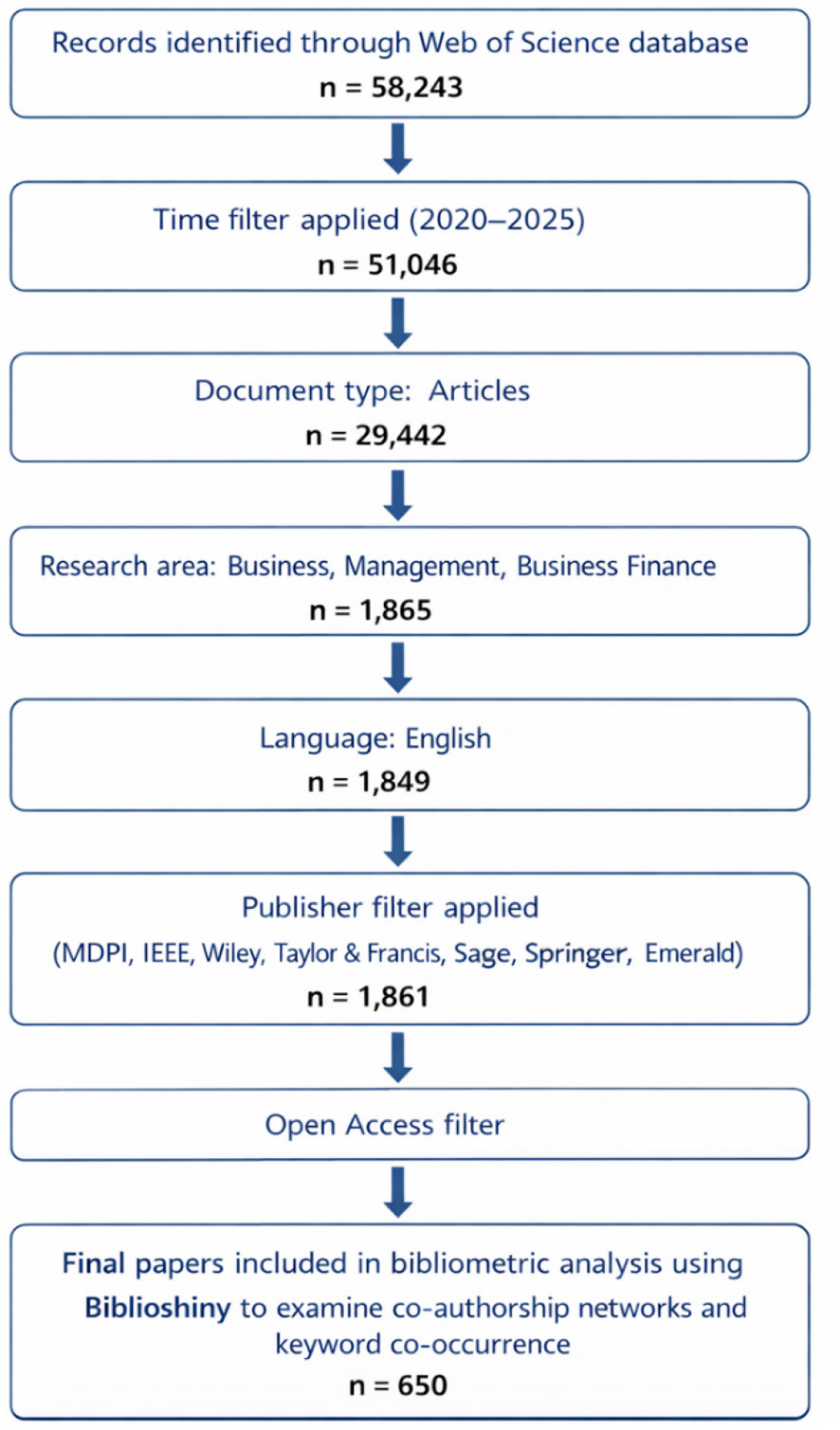
PRISMA flow diagram of article selection from web of science dataset.

The search performed in the Web of Science resulted in the retrieval of 650 records, with the help of the applied filters. These documents were filtered to only keep articles that were useful to artificial intelligence-based personalization and impulsive buying behavior within the framework of online consumer research. The Scopus database search yielded 48 records with the same key word strategy. Having examined the Scopus findings, it was identified that the dataset reflected a smaller but more related group of literature, with one article being duplicated with the records obtained with OpenAlex and Dimensions. This overlap further established the fact that the topic has restricted yet identifiable coverage in databases. Relevance, document type, language and subject were considered in the selection process based on the research objectives. Lastly, other filtering parameters such as relevance of publishers and access through open access were used, and the final data on 650 articles was used to carry out bibliometric analysis (Figure 1). The last datasets were thus utilized independently: Web of science as the primary dataset and Scopus as the comparison dataset.

### 2.4 Data analysis

Bibliometric analysis was performed by Biblioshiny, which is the web interface of Bibliometrics package in R. To make the two datasets comparable, the same procedure was undertaken in their analysis. The performance analysis was done in the first place to investigate the annual scientific production, the sources of key sources, the productivity of authors and the contribution of institution and countries. Second, the literature was examined with science mapping methods to examine the co-occurrence of keywords, thematic organization, and conceptual relationships. Third, the Law of Bradford and the Law of Lotka were used to determine the patterns of source concentration and productivity of authors, respectively. In the case of the Web of Science data the analyses were the major mapping of the field. The same steps were repeated on the Scopus dataset to check the presence of similar structural patterns in a smaller corpus. Two datasets were then compared at descriptive level to determine the consistency in the key themes, sources and collaboration patterns. The further searches in OpenAlex and Dimensions were conducted as validation checks only and were not regarded as independent analysis corpora since they retrieved a single article each, and the article was already present in Scopus.

### 2.5 Reliability and validation

The topic was cross-tabulated to enhance the strength of the study by conducting a search in several databases with the same key-word strategy. The scanty further retrieval on OpenAlex and Dimensions indicated that the topic is very narrow and unevenly represented on sites. In line with this, the study construed the findings as a bibliometric mapping of a newly emerging field of study as opposed to a full survey of the available literature. This method enhances the clarity of the analysis and consideration of the boundaries of database coverage.

## 3 Results

The bibliometric analysis relies on two datasets, using Web of Science as the primary corpus and Scopus as the second one of the comparison data. The Web of Science database covers 650 articles published in 2020–2025 and found in 172 sources, which means that the studies on AI-driven personalization and impulsive buying are distributed by a wide interdisciplinary literature, but not concentrated in a single established area. The spread of sources indicates that the subject is directly related to digital marketing, consumer behavior, information systems and data analytics, which make up the intellectual context of this new area of research.

A collaborative structure is also high in the Web of Science corpus. The dataset consists of 1,985 authors, only 60 single-authors articles and a mean of 3.43 co-authors per document with an international co-authorship rate of 52.77. These signs indicate that the discipline is evolving with multi-author and cross-border research collaborations, indicating the technical and behavioral intricacy of researching AI-based consumer impact on digital commerce. Also, the 2,240 author keywords and annual growth rate of 12.67 are additional indications that the literature is conceptually rich and growing at a very high rate, especially over the past years. The age of the documents (2.24 years) also suggests that the majority of the studies are not old as they are still a young and rapidly evolving field of research.

The Scopus database offers a smaller yet valuable comparison corpus having 48 records obtained by the same key-word strategy. The Scopus results, which remain far smaller, broadly follow the same pattern of development as Web of Science, with increased publication growth, and focus on a few sources, and again with a focus on AI, consumer behavior and impulsive buying. The smaller corpus does verify that the topic is represented throughout databases but also reveals that the coverage of the topic remains uneven and remains limited in certain indexing platforms. This reinforces the suggestion that the discipline is still immature and not completely developed.

**Figure 2:**
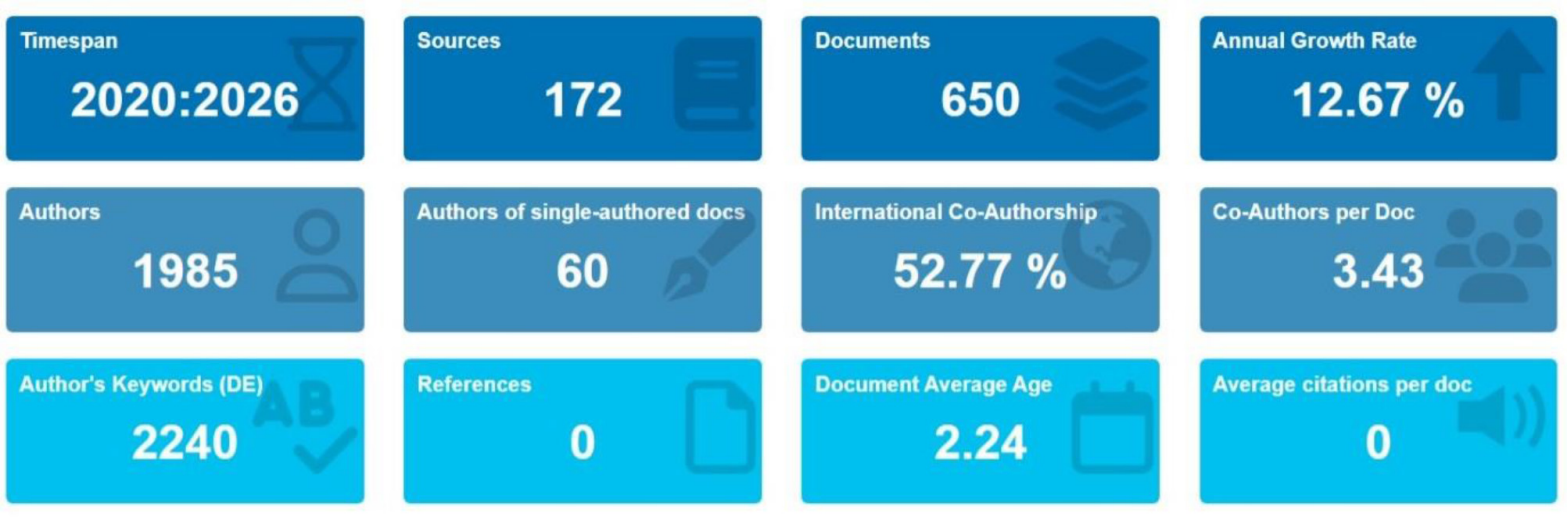
Overview of bibliometric dataset from WOS.

Combined, the two sets of data give a better idea of the research landscape. The Web of Science corpus provides the wider perspective of the area whereas the Scopus corpus is used as the validation layer which helps to support the main trends which we see in the bigger dataset. In both databases, the evidence is directed to an interdisciplinary, collaborative and fast-growing field of research, and more focus on the effects of AI-driven personalization on consumer decision-making and impulsive purchasing behavior in an online setting (see Figure 2).

### 3.1 Descriptive overview of publications

The Web of Science dataset on the annual scientific production presents a clear trend of increasing research on the topic of AI-driven personalization and impulsive buying behavior throughout the period of study. During the initial years, the rate of publication was low meaning that the subject matter was still in the initial stages and had not been given serious scholarly focus. Since the later years, however, the number of studies has been increasing more consistently, with the most recent period showing the most significant increase. This trend indicates that the subject matter has slowly transformed into a niche field of study to one that is now becoming more prominent in the digital commerce, consumer behavior, and AI-related study. It is also indicative of an increasing place of algorithmic recommendation systems, personalized marketing, and predictive analytics in influencing online consumer decision making, particularly among Millennials and Generation Z. The general direction is the same as in the Scopus dataset, albeit on a considerably smaller scale. Despite this limitation in the size of the corpus, the annual output is an indication of recent interest in the topic, with publication activity being focused on the later years of the period, as opposed to the earlier part of the period. This supports the assumption that the theme exists in databases, but that Scopus covers only a limited portion of the literature in this incredibly specific research field. The less important number of records also signifies that the theme is not yet popularized on all bibliographic platforms, which can be attributed to an emerging field instead of a well-developed one. Nevertheless, the Scopus trend helps to observe the opinion that the scholarly interest to AI-based personalization and impulsive purchase has become recentered and more focused.

**Figure 3:**
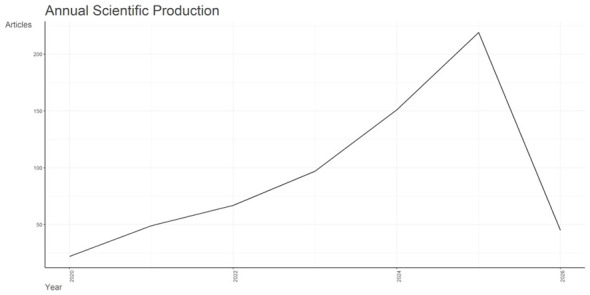
Annual scientific production from WOS dataset analysis.

Collectively, the two datasets demonstrate that the field is in its infancy and is progressing in the right direction, with Web of Science providing the bigger picture and Scopus a smaller confirmation of the same trend. The overall intuition is that the studies of AI-grounded personalization and impulsive purchasing are becoming more topical, particularly concerning digitally active generational groups, and that it is gaining momentum due to the rising power of AI-grounded online shopping landscapes (see Figure 3).

#### 3.1.1 Core publication sources in the research domain

The publishing trend in the Web of Science database demonstrates a wide and clear intellectual framework within the area of personalization using AI and impulsive buying behavior in Millennials and Generation Z. Though the data consists of 172 sources, a comparatively small pool of journals has a significant share of the literature, which suggests that the subject gradually becomes structured around a handful of powerful sources of publications. One of the most notable contributors is the Technological Forecasting and Social Change, which emphasizes the strong correlation of this subject with the larger discussions about technological change and its effect on society. Similarly, the Journal of Theoretical and Applied Electronic Commerce Research indicate the key role of online shopping platforms, recommendation engines, algorithm interfaces, and customer-specific marketing in influencing online buying behavior. The Journal of Innovation and Knowledge also highlight the relevance of technological innovation in improving the consumer experience and helping to drive impulse purchases. Meanwhile, the topic is also supported by such journals like the Journal of Business Research and the Journal of Retailing and Consumer Services as well as by Tech novation, Industrial Marketing Management, and Psychology and Marketing that provide strategic, innovation-based, and behavioral approaches to the topic. All in all, the fact that the publications are in a comparatively small pool of interdisciplinary journals may indicate that the field in question is slowly becoming more organized and the fact that technology, marketing, and consumer psychology are all united in one research area.

The Scopus database indicates the same overall trend, albeit in a smaller, more compact format. The first source is Sustainability (Switzerland), which is followed by Lecture Notes in Networks and Systems, which means that the issue is being covered both in the form of a journal and a conference publication. The involvement of technology-oriented and marketing-oriented sources is reflected in the moderate contribution of ACM International Conference Proceeding Series and the Journal of Research in Interactive Marketing. The other sources such as Advances in Intelligent Systems and Computing and Agriculture (Switzerland) are each only adding one or a few articles, indicating that the area remains spread out in multiple interdisciplinary publication outlets. Despite the Scopus corpus being limited, it nevertheless supports the idea that a small number of sources are starting to have a more tangible effect on the literature on AI-driven personalization and impulsive buying.

**Figure 4:**
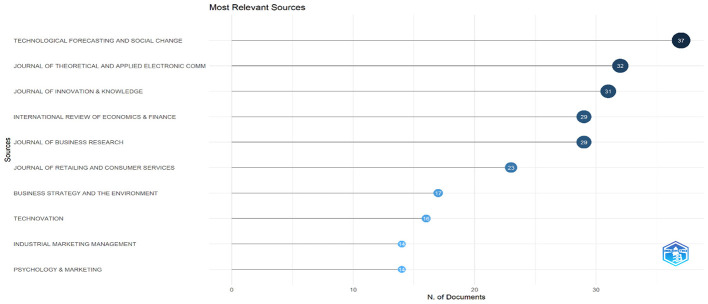
Relevant sources.

Combined, both data sets indicate that the literature is distributed over a variety of sources, yet few journals and series of publications are being centralized as sources. Web of Science presents the bigger and more established picture, whereas Scopus proves the identical trend on a smaller scale and demonstrates that the field of research is interdisciplinary, emerging, and increasingly focused on a limited number of influential sources of publication (see Figure 4).

#### 3.1.2 Source dynamics

**Figure 5:**
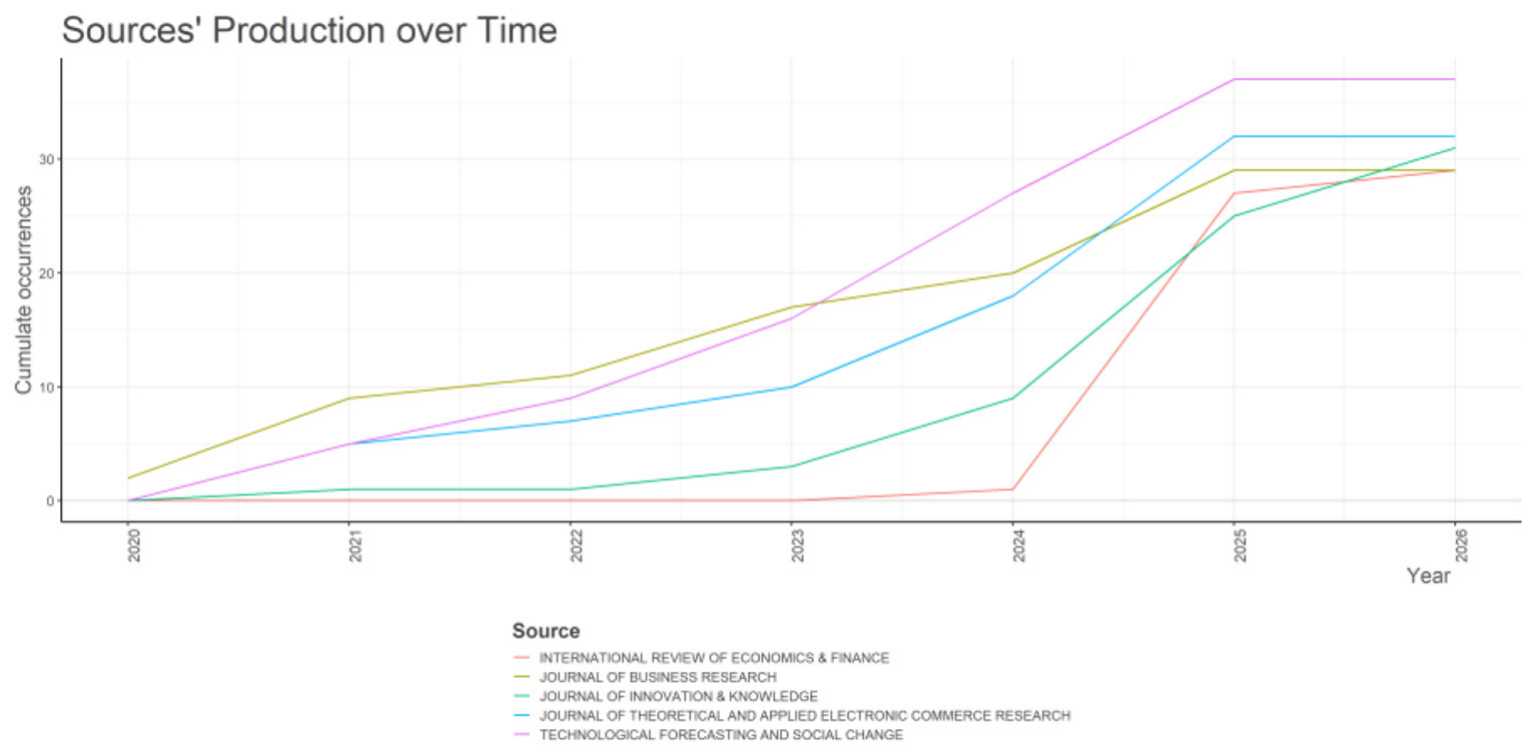
Production over time.

The following Figure 5 represents the contribution of major journals to the research on AI-based personalization and impulsive buying behavior over the period 2020–2025. Instead of presenting the total number of publications, the figure emphasizes the extent to which the main journals have over the years influenced the academic growth of this field. There is an apparent monopoly of the output in a few powerful journals, particularly those in Technological Forecasting and Social Change, Journal of Business Research, and Journal of Theoretical and Applied Electronic Commerce Research. These journals have a consistent cumulative increase, which is an indication that they are the center of knowledge building on the topic of AI-based consumer behavior and algorithmic personalization. Technological Forecasting and Social Change exhibits the best positive trend, indicating that AI-based personalization is under investigation in the wider framework of technological change and digitally driven market change.

The journal of Business Research demonstrates an enduring interest in consumer behavior and marketing analytics, which validates the importance of personalized systems as behavioral stimuli that may be used to affect impulsive buying. As demonstrated by the rising importance of e-commerce platforms as important environments of AI application, the relevance of the Journal of Theoretical and Applied Electronic Commerce Research is growing. Other journals (like Journal of Innovation and Knowledge) demonstrate a slower yet observable increase, indicating that AI personalization is also being considered as an innovation-driven phenomenon. The emergence and rise of journals like International Review of Economics and Finance in the recent past is also a pointer that the subject is gaining interest both economically and as far as marketing is concerned. In general, the trend of the growth of publication activity after 2023 indicates the growing role of Millennials and Generation Z in digital marketplaces, and demonstrates that the field is becoming more dynamic, interdisciplinary, and directly connected with the latest trends in AI-based commerce.

The chart of Scopus called Sources Production over Time demonstrates a smaller scale pattern. It shows that only a small number of sources started to contribute at the beginning but most of the sources came later, particularly after 2018, and the strongest growth came after 2020. Increase in the lines is in a stepwise fashion indicating that the productivity of the sources is concentrated in few outlets and that most sources are only intermittent contributors. Some sources have even higher growth rates in the later years, implying recent spurts of interest instead of more lasting domination. Collectively, the Scopus pattern confirms that the discipline is growing, although it is still disjointed and being influenced more by newer or even an *ad hoc* group of contributors than by a substantial pool of constant and consistent sources. Their joint knowledge is that the study of AI-mediated personalization and impulsive purchasing is becoming more and more focused in few major journals, yet continues to be open, interdisciplinary and dynamically developing (see Figure 5).

### 3.2 Source and author productivity

#### 3.2.1 Bradford's law analysis

The Law pattern of Web of Science data reveals a very concentrated academic system, in which a comparatively few journals provide a huge portion of the literature on AI-driven personalization and impulsive buying behavior. Instead of being randomly spread over numerous outlets, the publications are concentrated in a core set of highly productive resources, such as Technological Forecasting and Social Change, Journal of Theoretical and Applied Electronic Commerce Research, Journal of Innovation and Knowledge, International Review of Economics and Finance, Journal of Business Research, Journal of Retailing and Consumer Services, Business Strategy and the Environment, and Industrial Marketing Management. Such a focus shows that the intellectualization of the discipline is being propelled by a small number of key journals that have become central to the theoretical and empirical discourse around AI-based consumer personalization.

The Scopus data presents the same overall trend albeit in a smaller and more condensed format. The core zone of the literature is Sustainability (Switzerland) and Lecture Notes in Networks and Systems which means that they are the most fruitful sources in the Scopus corpus. The second zone of distribution of Bradford is moderate contributions made by ACM International Conference Proceeding Series and the Journal of Research in Interactive Marketing. Of these sources, the majority of outlets only add one article each, which demonstrates the peripheral location and proves the fact that the literature remains scattered over a broad interdisciplinary spectrum. This trend is greatly in favor of Bradford Law, because a few central sources are found to contribute a large quota of the production, and the other work is distributed in numerous less fruitful journals and proceedings.

**Figure 6:**
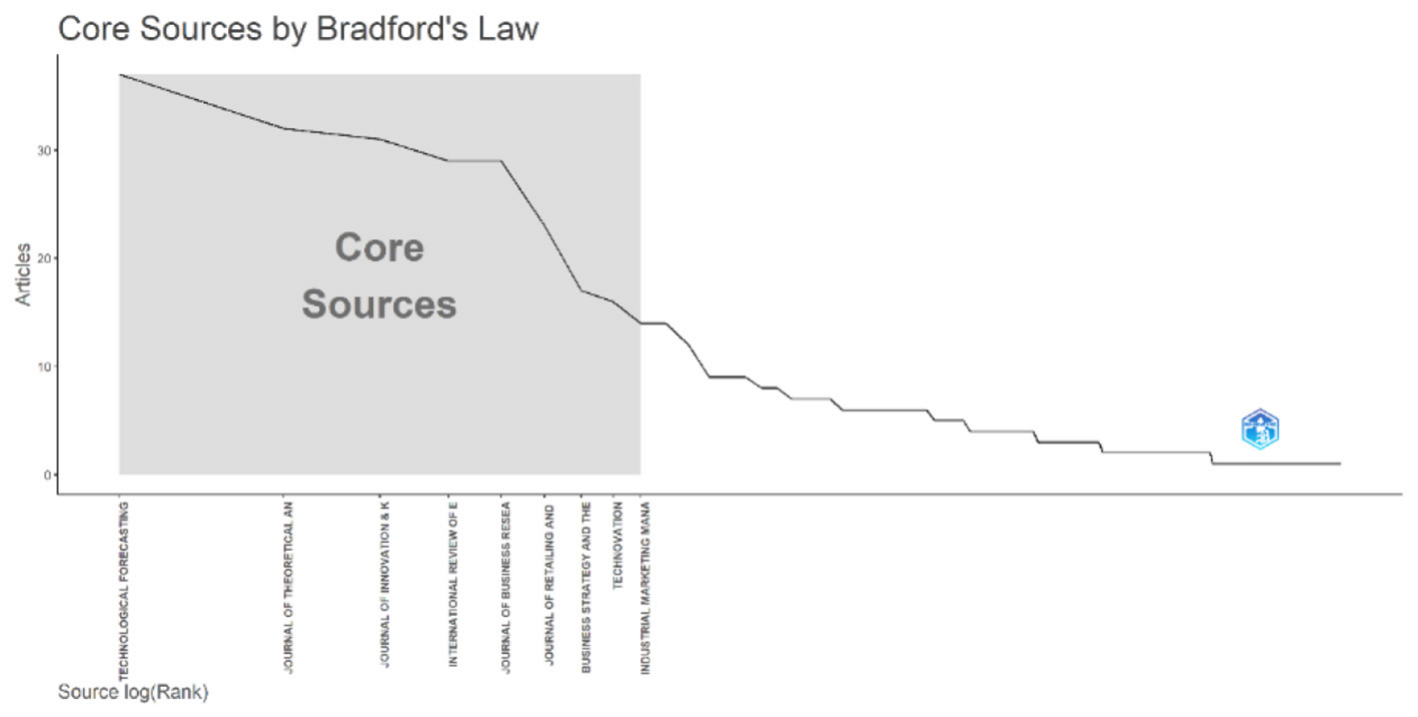
Bradford's law.

Combined, the two sets reveal that the data on AI-based personalization and impulsive purchasing is rooted in a few sources and is still in its nascent stage and not consolidated. Web of science gives us the more general and time-proven intellectual framework whereas Scopus validates the same general tendency at a smaller level. The synthesis of what has been learned is that the subject is inter-disciplinary, becoming more and more focused on major publication venues and continuing to develop in the literature, both journal and conference based (see Figure 6).

#### 3.2.2 Lotka's law analysis

**Figure 7:**
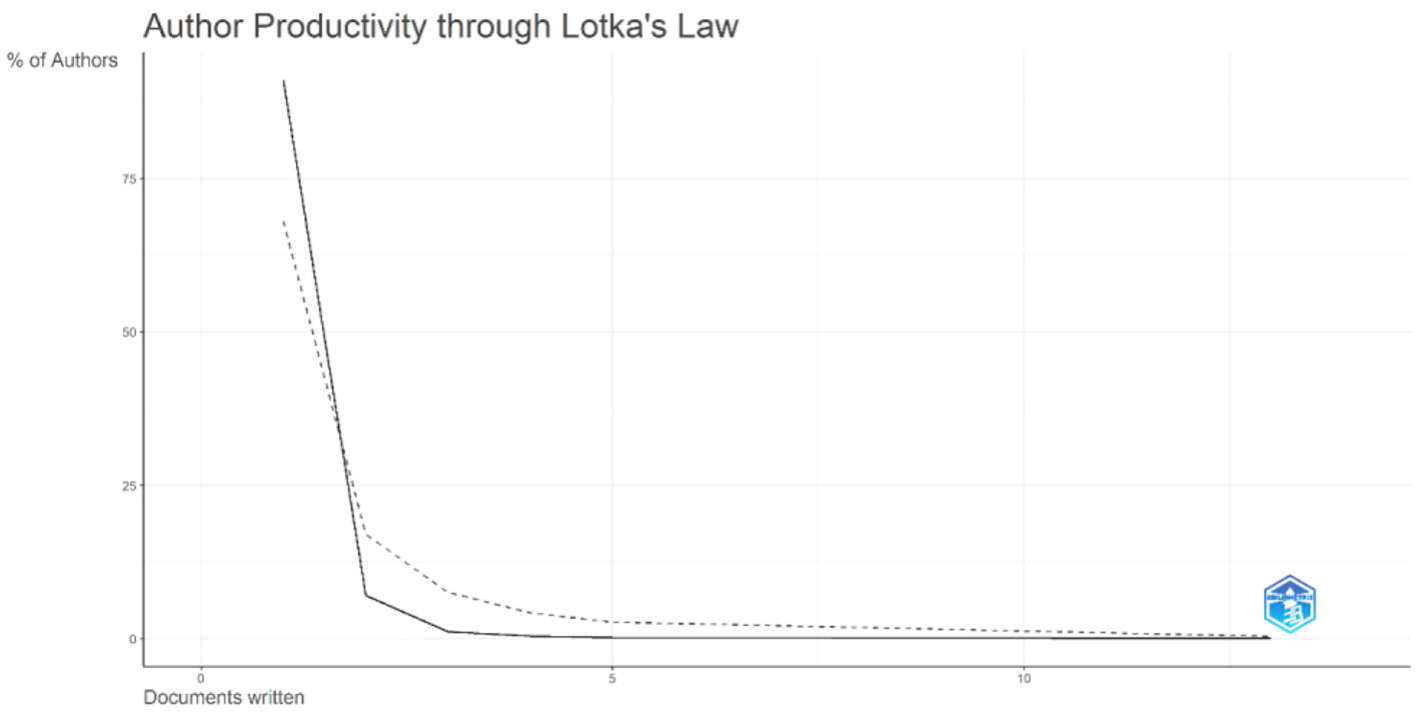
Lotka's Law.

The Figure 7 presented in the Web of Science Lotka demonstrates that productivity of authors in the sphere of AI-based personalization and impulsive buying is extremely uneven with the majority of authors contributing to the field only once and a very limited number of authors publishing repeatedly. The sharp decline in the empirical curve relative to the theoretical Lotka distribution suggests that there is a high volume of authors in the field, but a dispersed one, which is characteristic of an interdisciplinary area that is emerging and seeks to use marketing, information systems, innovation management, and data analytics. This trend also indicates that the literature remains in its infancy and that the creation of knowledge is still in the state of many autonomous contributors and not in the hands of a few stable research groups. Within the framework of your Millennials and Generation Z research, this, also, reflects a gap: a great part of the literature seems to consider younger digital consumers as a subset of larger samples, not as an analysis target.

The result of the Law of Lotka in Scopus dataset also demonstrates a similar overall structure, but more compact. The number suggests a high rate of single author contributions, and a much smaller number of two and more contributions, which is expected with an emergent and interdisciplinary field. The distribution is not very far off, and this is indicative of the fact that the subject of study remains largely reliant on occasional contributors as opposed to having a big number of highly prolific scholars. This implies that the field is still emerging and has not yet established a thick and specialized community of authors that is consistently interested in AI-based personalization and impulsive purchasing.

Combined, the two datasets demonstrate that the field of research is dynamic but continues to be disjointed authoritatively. Web of Science proposes a less concentrated prolific author distribution than would be the case with Lotka model, whereas Scopus displays a more regular (yet still constrained) structure of productivity. The collective understanding is that the discipline is expanding, interdisciplinary, and yet remains intellectually open, yet lacks a coherent core of returning scholars to continue to add to the same research stream, particularly with reference to Millennial and Generation Z consumer behavior.

### 3.3 Geographic distribution of research

#### 3.3.1 Country scientific production

The geographical distribution of the scientific output of the Web of Science dataset indicates that the study of AI-based personalization and impulsive buying behavior is concentrated in few technologically advanced countries. The most fruitful contributors are China and the United States, which are the most developed artificial intelligence ecosystems, have the largest e-commerce markets, and can access digital consumer environment where the study of personalized marketing can be conducted empirically. The second ring of activity is in the countries of the United Kingdom, Germany, Spain, Italy, India and Australia, which indicates that the subject is also on the rise in Europe and the Asia-Pacific region. This trend suggests that the literature is directly associated with areas that have a well-developed academic system, strong digital economy, and prevalence of online shopping and recommendations systems.

The Scopus data reveal a more limited distribution of the research activity. China and India are the primary contributors, with the United States being relevant as well. Moderate levels of publication activity have been observed in several countries across Europe, Southeast Asia, and Australia, and it helps to confirm that the topic under consideration is being researched primarily in the areas with well-developed digital commerce ecosystems and growing research potential in AI and consumer behavior. Meanwhile, significant portions of Africa, as well as parts of South America, are still underrepresented, with an apparent geographical imbalance in the literature. This indicates that the discipline continues to be dominated by a comparatively few nations and that academic involvement by underexplored areas is minimal.

**Figure 8:**
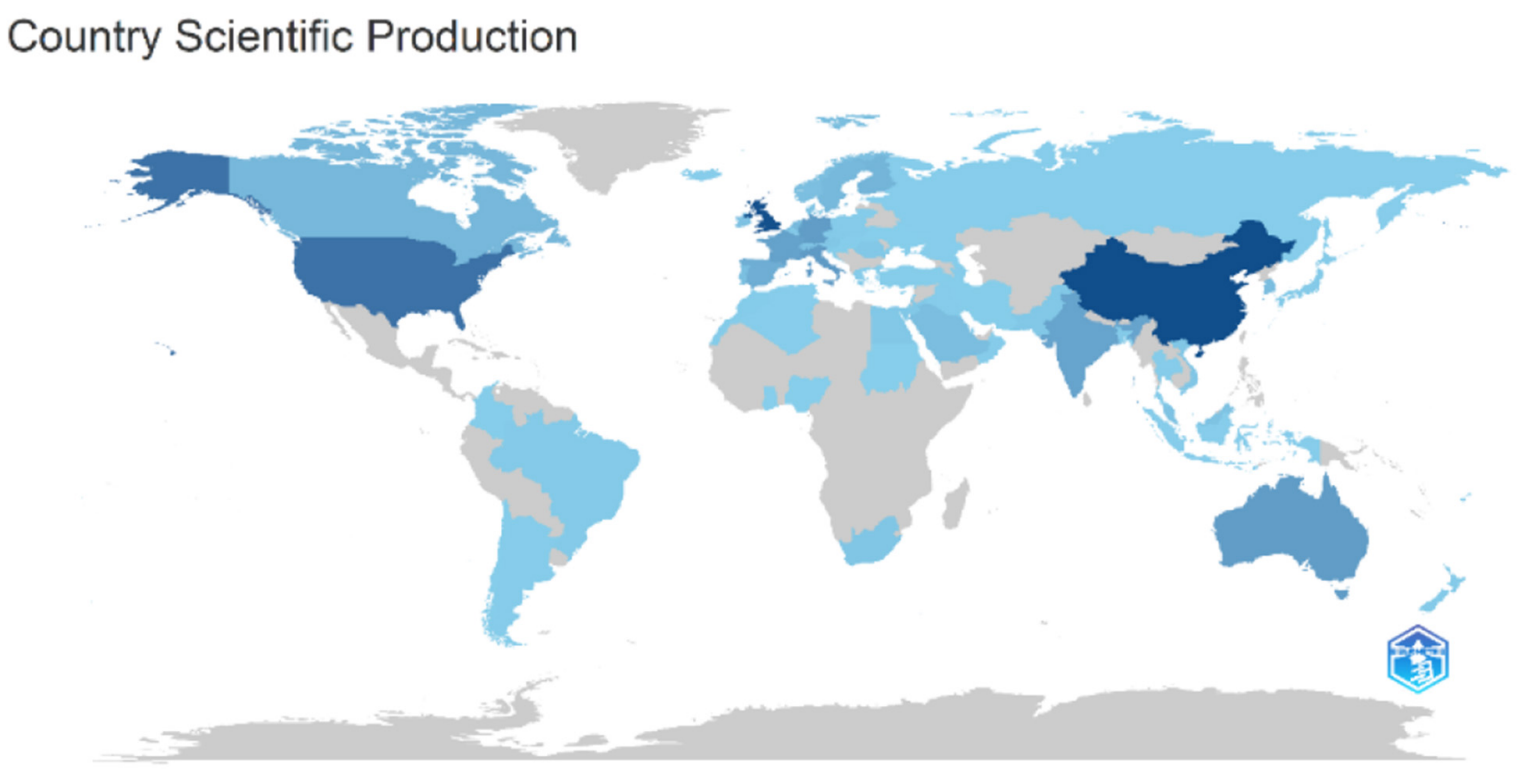
Country scientific production.

When combined, the two datasets indicate that the geography of research is distributed all over the world but unevenly distributed. Web of Science reveals the larger trend of leadership by China, the United States and other developed economies, and Scopus validates the larger trend on a smaller scale, with China and India being particularly outstanding. The synthesis is that AI-based personalization and impulse purchasing is a global research focus though the most significant academic interest remains in those countries with well-developed digital markets, and there is still a range of opportunity to do research in the underrepresented regions and conduct cross-cultural research on Millennial and Generation Z customers (see Figure 8).

#### 3.3.2 Country production over time

The trend of country-production in the Web of science dataset is characterized by the stable increase in the level of scientific activity in the leading countries during the period of 2020–2026, and then the sharp increase in the last few years. This growth indicates the fast growth of artificial intelligence usage in online business, particularly recommendation engines, predictive consumer analytics, and personalized marketing engines. China and the United Kingdom are the most prolific countries represented, meaning that both are key to scholarship in the field. The United Kingdom presents a sustainable and steady growth trend, which is consistent with its high academic standards in marketing analytics, consumer behavior, and online commerce, whereas China presents recent rapid growth, probably nurtured by its substantial e-commerce infrastructure and substantial investment in AI technology. The contribution in the United States is also very high, and this is because of its technological innovation, marketing research and consumer analysis that is data driven. Germany, Italy, and Australia have moderate, albeit steady growth, which indicates that more countries are taking part in AI-enabled consumer behavior studies. Generally, the Web of Science trend indicates that studies concerning AI-driven personalization and impulsive purchasing are on the increase, yet they are still limited to technologically advanced economies, in which the level of digital infrastructure and e-commerce is well-developed.

The country-production chart of the Scopus indicates a similar, though dynamic trend. China is at the top of the cumulative growth with the strongest and most sustained, with an early contribution and a sharp increase after 2021. India, as a latecomer, demonstrates the highest growth rate after 2023 and is among the global leaders in the research destination by 2026, which suggests its fast becoming one of the largest research destinations in the field. The United State has quite stable yet less vigorous growth, and its trend is upwards throughout the time. The other nations like South Korea, Indonesia and Slovakia are later in the history and add smaller but steadily growing amounts, which implies that the subject is growing beyond the first group of dominant nations. This trend suggests that the more conspicuous, geographically expanding research space is encompassed by Scopus, and India is becoming prominent among other countries in addition to China.

**Figure 9:**
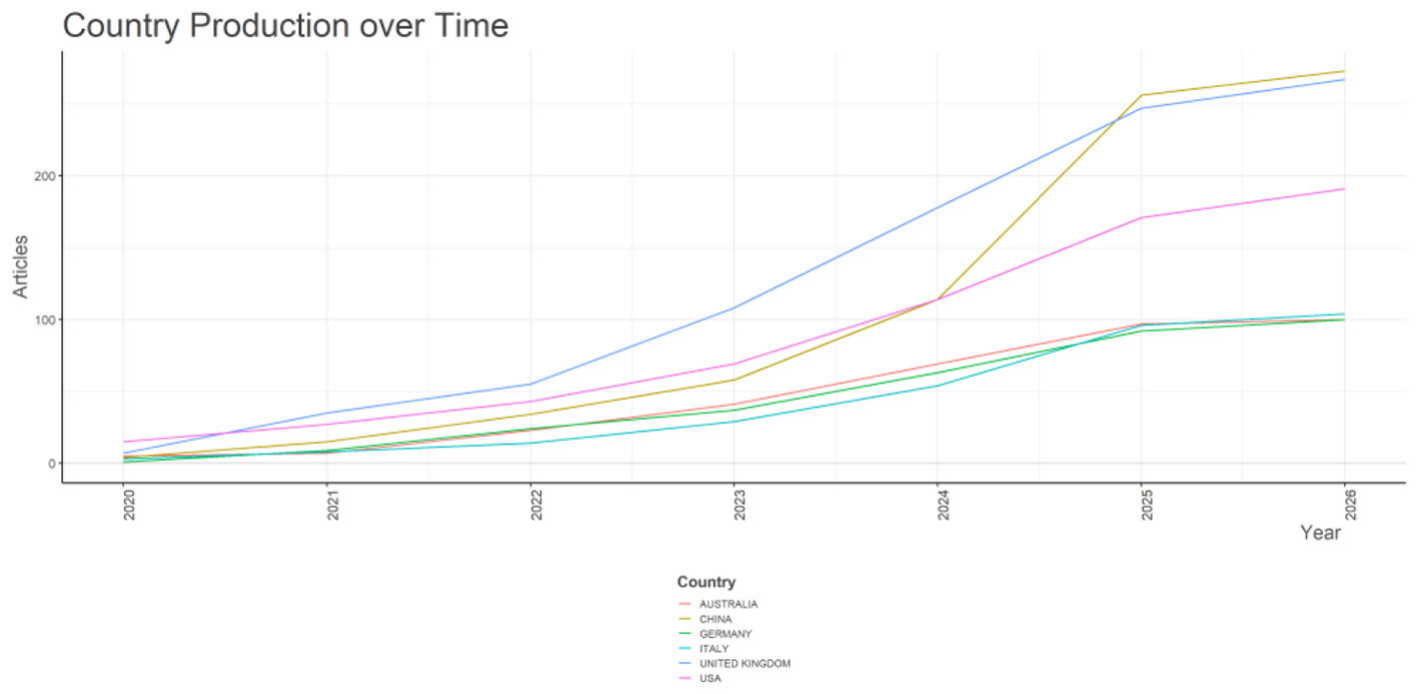
Country production.

Combined, both sources indicate that the geographical expansion of the research on AI-driven personalization and impulsive buying is also limited to a limited number of top countries, yet the sphere is also expanding with time. Web of science gives us a bigger picture of the continued leadership by China, the United Kingdom, and the United States whereas Scopus gives us the picture of the escalated leadership of India and the increased involvement of other nations. The cumulative takeaway is that the subject is gradually turning into a global issue, but the best research growth is still found in nations that have developed digital economies, high levels of AI development, and a well-established e-commerce infrastructure (Figure 9).

#### 3.3.3 Global scientific collaboration network

The map of country collaboration in the dataset of the Web of Science reveals the international system of research cooperation related to AI-powered personalization and impulsive purchasing. Instead of merely demonstrating the geographical sources of publications, the map demonstrates the way of knowledge exchange among regions and how research cooperation is structured with the help of large international centers. In North America, Europe and East Asia, three powerful integration centers can be seen, where the United States plays a central role in linking various research centers. This is an indication that the United States is a key intermediary in promoting interdisciplinary studies on AI in consumer behavior and online shopping. China also emerges a strong center and has close relationship with advanced research market and economies with high technology. It is a powerful collaboration in the analysis of algorithmic recommendation systems, personalized advertisement, and impulsive buying behavior due to its huge e-commerce ecosystem and fast growth in artificial intelligence. Another close collaborative network is the one between the United Kingdom and other European nations, which indicates a high level of relations in digital marketing, consumer analytics, information systems, and the science of behavior. This tendency indicates that the European cooperation is particularly applicable in researching the psychological and strategic aspects of the AI-based personalization.

The Scopus collaboration map represents a more or less similar map of international research networking. China, the United States and India emerge as key players, with apparent ties, which reflect ongoing cross-border cooperation. China is once again at the center stage indicating its high output of research and its lead in the field. The linkages between the U.S., Europe and Asian nations are indicative of a very interconnected research ecosystem where knowledge sharing is taking place between regions. The new involvement of countries like Brazil and Australia indicates that the field is more geographically varied and more global in nature. This means that AI and consumer behavior research has no geographical constraints but is growing by collaborating internationally. In the case of research on Millennials and Generation Z, this is relevant since digitally native consumer groups are spread in a plethora of cultural and economic conditions and comparative research is particularly beneficial.

**Figure 10:**
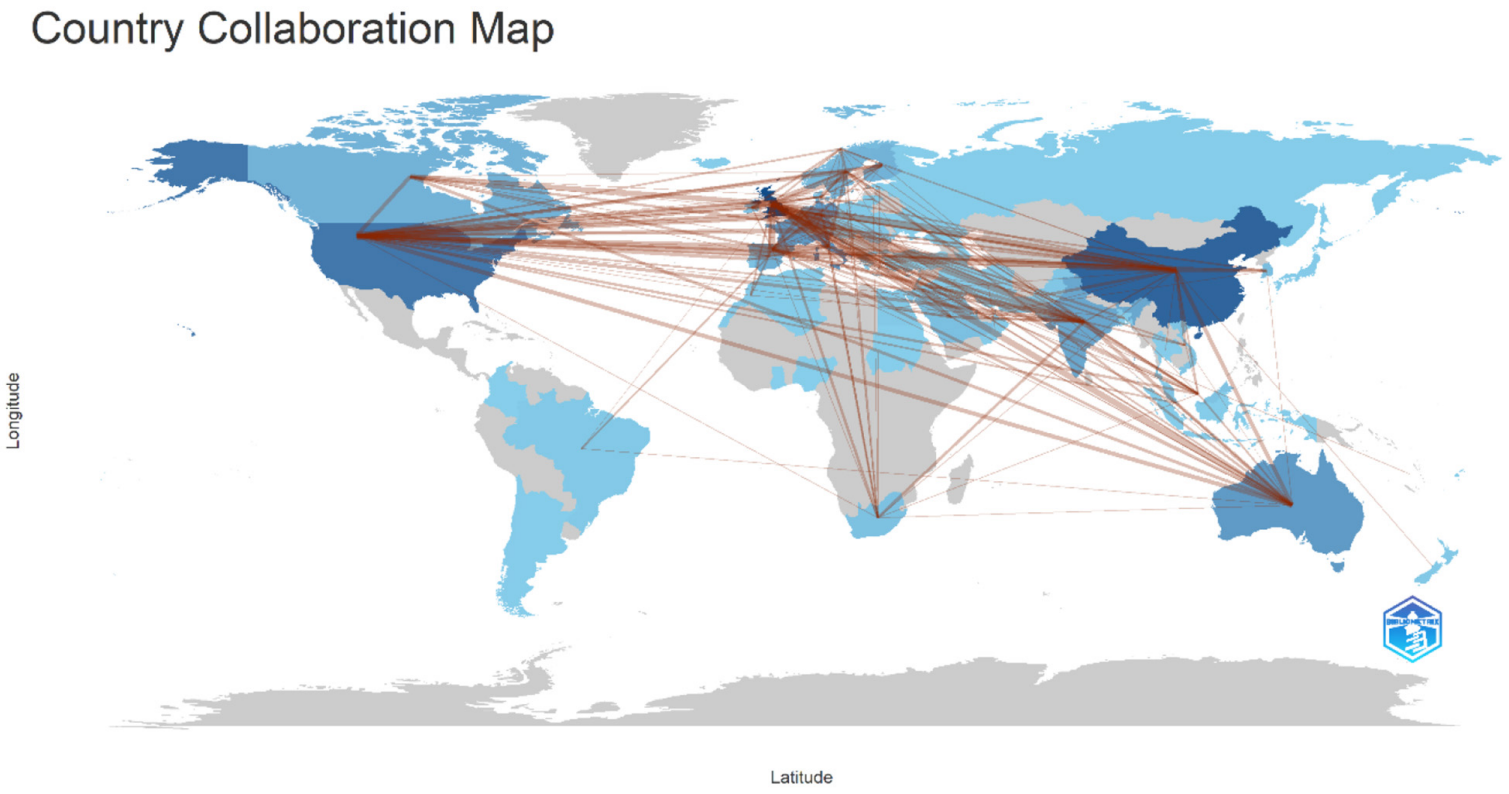
Country collaboration map.

Collectively, the two datasets reveal that the studies of AI-powered personalization and impulsive purchasing are getting more global. Web of Science emphasizes the most powerful hubs of collaboration and the center stage of the United States, China, and Europe, whereas Scopus demonstrates a broader distribution of collaboration including India, Brazil, and Australia. The collective wisdom is that the field is no longer limited to nationalized research systems, rather it is being influenced by cross-regional collaboration, which enhances methodology heterogeneity and presents comparative research possibilities across the various digital economies and generational markets (See Figure 10).

### 3.4 Keyword and conceptual structure

#### 3.4.1 Most relevant words

The Web of Science keyword analysis shows that the research on AI-driven personalization and impulsive buying is conceptually centered on Artificial Intelligence, which appears as the most dominant keyword. This indicates that the literature mainly frames impulsive buying behavior within a broader technological environment shaped by AI-driven digital systems rather than treating it only as a psychological buying tendency. Other frequent keywords such as technology, AI, and big data show that the field is strongly tied to data-driven consumer analytics, recommendation systems, and algorithmic decision support. These terms reflect the growing role of digital platforms in using consumer data to predict preferences and deliver personalized stimuli that may encourage impulse buying.

At the same time, keywords such as management, innovation, and performance show that the field is not limited to technology alone. Instead, it also includes strategic and organizational perspectives, suggesting that AI-based personalization is increasingly viewed as a tool for improving customer engagement, platform effectiveness, and business performance. In the context of Millennials and Generation Z, this is especially important because these cohorts are digital natives who interact frequently with personalized online environments. The keyword pattern therefore suggests that the Web of Science literature connects AI technologies with behavioral marketing and strategic management, highlighting the relevance of AI-driven personalization in shaping impulsive purchasing behavior on modern e-commerce platforms.

The Scopus collaboration map, by contrast, shows the international structure of research partnerships, with China, the United States, and India emerging as major contributors. The visible links between the United States, Europe, and Asian countries indicate a highly interconnected research network, while the participation of Brazil and Australia suggests that the field is gradually becoming more global. Taken together, the Scopus map shows that AI and consumer behavior research is no longer confined to a single region but is expanding across multiple digital economies, creating opportunities for comparative and cross-cultural studies.

**Figure 11:**
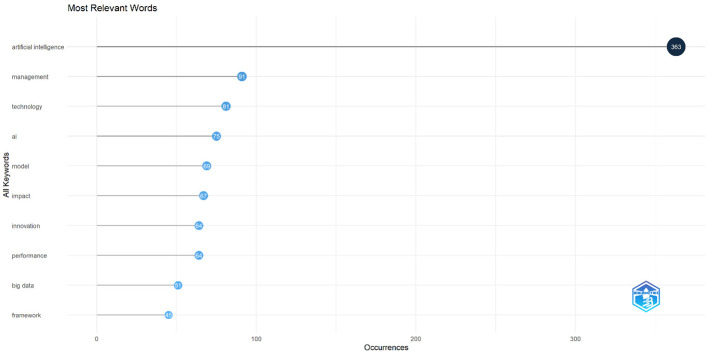
Most relevant words.

The combined insight from both datasets is that the field is both conceptually concentrated and geographically expanding. Web of Science shows that the literature is built around AI, technology, and data-driven personalization, while Scopus shows that the research community is becoming more internationally connected. Together, they suggest that AI-driven personalization and impulsive buying is a growing interdisciplinary field shaped by technological concepts, strategic business concerns, and cross-border collaboration (see Figure 11).

#### 3.4.2 Tree map analysis

As illustrated in the Web of science tree map, the conceptual center of the sphere is firmly rooted in the sphere of Artificial Intelligence that takes the biggest share and, thus, is the most influential concept in the literature on the AI-driven personalization and impulsive buying. This means that the sector is largely constructed on the technological advancement, particularly algorithmic recommendation engines, predictive analytics and automated decision-making tools that enable personalized online communication to occur. Very similar words like technology, AI, big data, and machine learning strengthen this technological orientation as much of the research revolves around the data-driven infrastructure that is at the back of personalized marketing. These ideas demonstrate the process by which online platforms analyze consumer data and provide customized recommendations, which can potentially trigger spontaneous purchasing behavior.

Concurrently, another significant shift is consumer-centered and managerial themes that can be also observed in the tree map. Such keywords as trust, adoption, decision-making, consumer behavior, and engagement show that the recent research is not limited to the technical aspect of AI, but also to consumer attitude to such systems. This is of particular importance in the face of Millennials and Generation Z, as these generations are the most active users of digital platforms and more exposed to AI-driven personalization in the context of shopping in a regular setting. The tree map thus implies that the literature is shifting toward a more balanced point of view, with the impact of AI on consumer experience and impulsive purchasing taking an equal role as to the technologies themselves.

The Scopus tree map, however, is more market-oriented and structured. The prevalent ones are the themes of artificial intelligence and consumer behavior with the next one being electronic commerce and marketing, which indicates that the area is highly focused on the interaction between the AI and the digital commerce. The middle categories like online consumer behavior, impulsive buying, machine learning and sales indicate that more and more focus is paid to how technology influences purchasing decisions. Smaller, yet still noticeable themes, like those of recommender systems, sustainability, personalization, data mining, and chatbots, suggest newer and more specialized areas of investigation. What it means is that the literature on Scopus is expanding and yet it has been pegged on a small number of central themes concerning digital business and consumer response.

**Figure 12:**
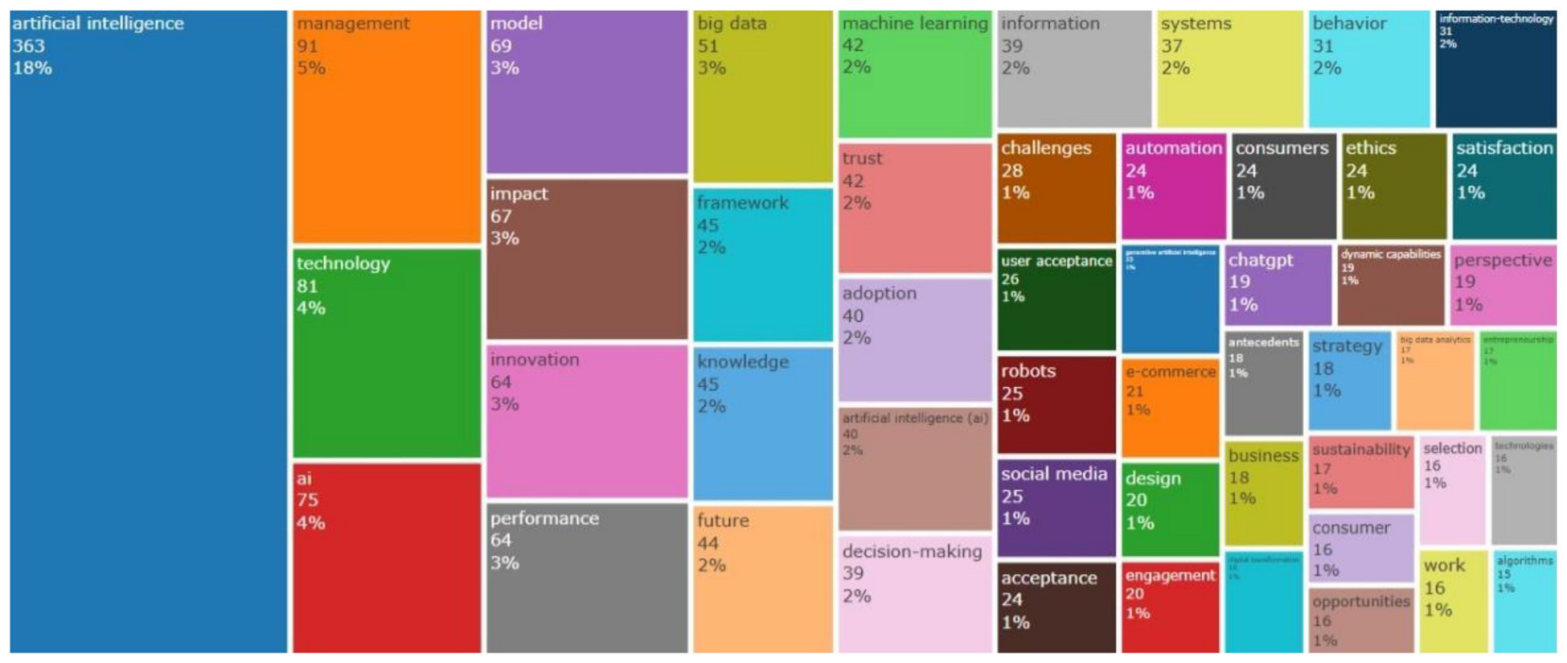
Tree map.

Combined, the two datasets demonstrate that the discipline is constructed on the overlap of AI and consumer behavior, although each database points out a bit of a variation on the focus. Web of Science emphasizes more on the technological underpinning of personalization whereas Scopus provides better visibility into the themes of consumer behavior, e-commerce and marketing. A synthesis of this insight is that AI-based personalization and impulsive purchasing is a fast-evolving interdisciplinary domain, where fundamental focus is on technology and consumer behavior, and with increasing diffusion to more specific and applied research domains (see Figure 12).

#### 3.4.3 Word frequency over time

This is demonstrated by the keyword-frequency trend in the Web of Science, which indicates that the intellectual emphasis of the discipline has been redirected toward artificial intelligence over the years. During the years of the study, the frequency of the keywords was low in the early years of the research, which indicates that the research on the topic of AI-driven personalization and impulsive buying was in its infancy. Since approximately 2023, though, the rate of occurrence of the term Artificial Intelligence skyrockets, and it thus becomes the most central element in the literature. Other terms that are in a similar vein like AI, technology, model and big data are also on the rise steadily, indicating that the area is becoming more and more firmly based on data-driven and algorithm-based consumer behavior on online markets. This trend is indicative of the increasing role of predictive analytics, machine learning, and recommendation algorithms in the creation of tailored online shopping experiences.

Simultaneously, the number of words, including the terms, like the notions of innovation, management, impacts, and performance, also grow, which implies that the literature is leaving the purely technical issues behind. These notions suggest that the strategic and organizational implications of AI implementation are also on the agenda of researchers, particularly, the impact of AI-based personalization on consumer reactions, marketing results, and the platform performance. The corresponding increase in the parallel of consumer behavior also demonstrates that the research is increasingly becoming behaviorally oriented, with increased focus to the influence of technology on the purchasing decisions. This is particularly applicable in the Millennial and Genz context since these digital-native customers are highly sensitive to the online environment which is highly exposed to AI-driven recommendation systems and personalized online experiences, which can affect impulsive buying behavior on-the-fly.

The Keyword-frequency pattern of the Scopus database indicates a more consumer-oriented development. There is a reduced activity in the field as there are very few keywords used in the early years. Since about 2018, the terms of artificial intelligence, consumer behavior, and online consumer behavior start to spread, with a slow increase in the research base. The frequency of several keywords, particularly, the keywords of vital importance, such as artificial intelligence and the consumer behavior, sharpens after 2022 and becomes the leading themes. Other keywords such as machine learning, electronic commerce, impulsive buying, increase steadily whereas sales and buying behavior increase more slowly. This shows that the Scopus literature is now focusing more on the connection between AI technologies and consumer decision-making when in an e-commerce context.

**Figure 13:**
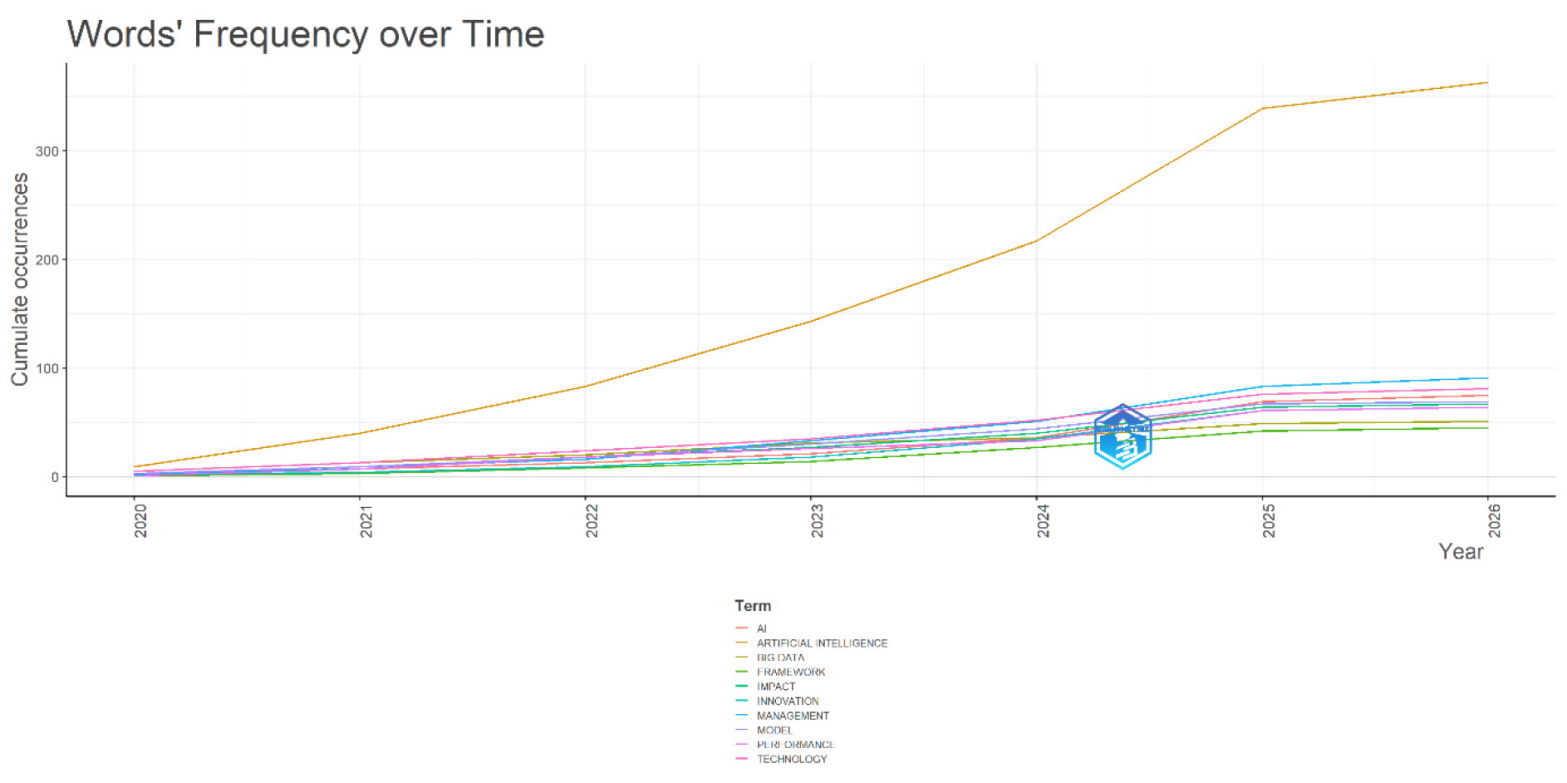
Word frequency.

Combining both datasets is an indication of a strong move toward consumer studies that are technology driven. Web of Science focuses on the increasing conceptual hegemony of AI and the strategic meaning of personalization, and Scopus gives prominence to the fast development of consumer behavior, e-commerce, and impulsive purchasing as a major research topic. The synthesis of these two is that the domain is growing rapidly, and AI is now serving as the primary intellectual foundation and consumer response becoming the most important behavioral consequence in digital commerce research (see Figure 13).

#### 3.4.4 Keyword co-occurrence network

The keyword co-occurrence network of the Web of Science reveals that the literature is structured around two significant thematic clusters that are closely related. Technological core of the field is the first cluster, focused on the concepts of artificial intelligence, and closely related terms include the machine learning, big data, algorithms, digital transformation, management, and technology. This collection indicates that the technical architecture that facilitates the personalization of AI-based systems on digital platforms is of primary concern in much of the research. The second cluster is consumer-focused and comprises of terms like e-commerce, consumers, behavior, trust, engagement, satisfaction and user acceptance. It means that it is significantly concerned with consumer-AI connection and is keen on understanding the influence of behavioral and psychological factors on online decision-making. Their close interconnection reveals that the sphere is constructed on the interplay of technological innovation and the consumer behavior particularly when it comes to impulse buying in the context of Millennials and Generation Z.

A similar, finer differentiated network is shown in the conceptual structure map on Scopus. The biggest and most interconnected term is situated at the center of the map and is called artificial intelligence, which indicates that it works as the driving force behind the research field. It is closely associated with the consumer behavior, purchasing, sales, machine learning as well as the decision-making process that represent a compact core group. There are a few sub-themes that are emanating around this central theme. One of them concentrates on online consumer behavior, e-commerce, online consumers, and marketplaces, indicating that digital commerce is a significant environment of AI-related studies. A second cluster encompasses the social media, sustainability, and consumption behavior, as well as the marketing, and is associated with general marketing and societal issues. Other peripheral nodes like data privacy, recommendation agents and online consumer behavior point to new but less well-linked themes, whereas impulse buying behavior is rather isolated, meaning that it remains not linked to the wider network of AI research.

**Figure 14:**
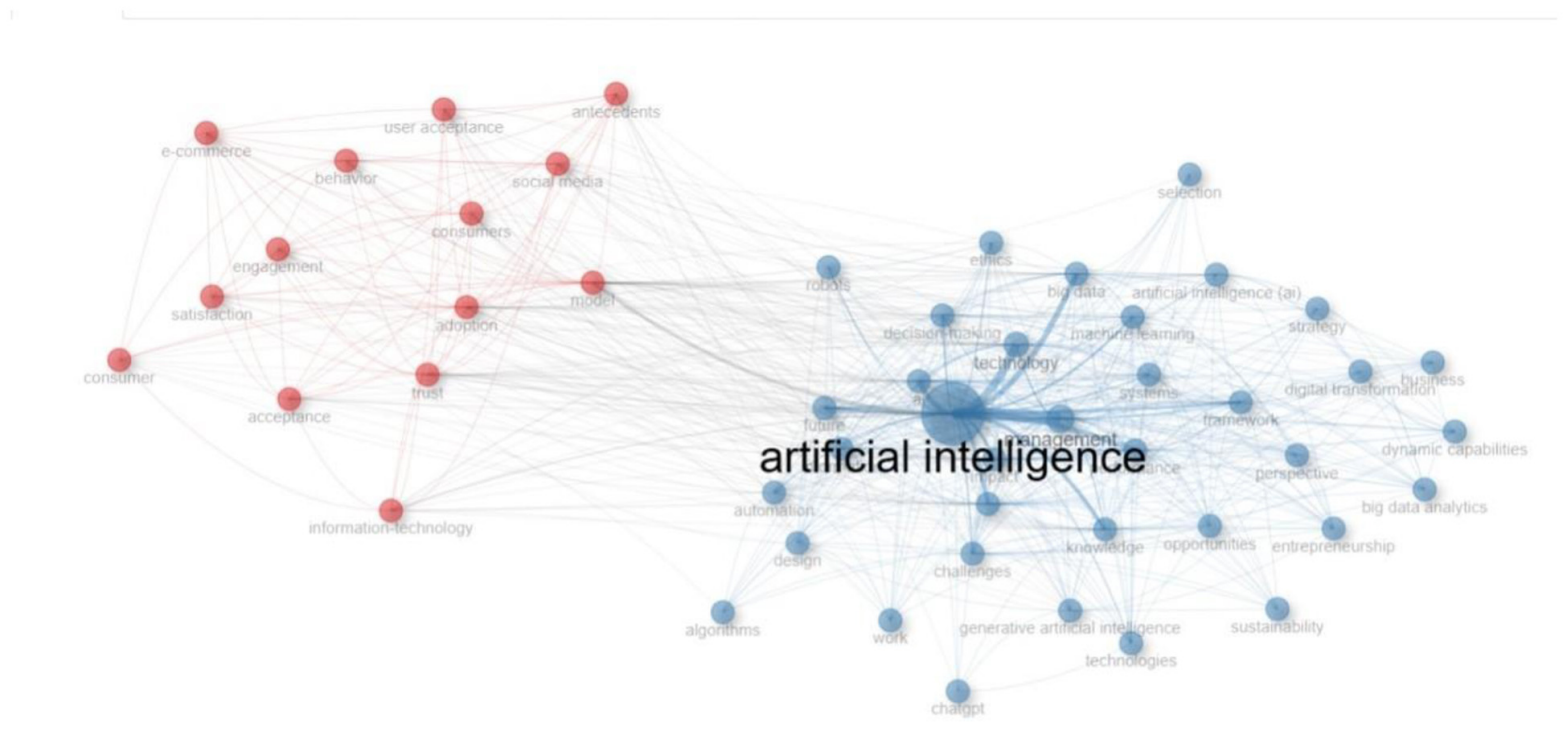
Keyword co-occurrence network.

Combined, the two datasets provide evidence that the field is heavily research-focused on artificial intelligence and its utilization in consumer behavior, yet they also indicate a significant gap. Web of Science brings out the general two cluster nature of technology and behavior, whereas Scopus demonstrates that, the impulsive buying behavior is still very peripheral even though it is very pertinent to the topic. The synthesis of these two ideas is that AI-driven personalization, combined with impulsive buying, is at the nexus of a prevailing technological mainframe and a weakly linked behavioral theme and thus an interesting field to research in the future (see Figure 14).

### 3.5 Emerging research topics

The Web of Science topic-trends analysis shows a clear time-based evolution of the field from early work on predictive analytics, customer experience, and attitudes toward more technically advanced themes. In the initial phase, the literature was mainly concerned with understanding how data analytics and consumer perceptions shape online purchasing behavior. As the field developed, topics such as big data, machine learning, and e-commerce became more prominent, showing a gradual shift toward algorithmic systems that process large volumes of consumer data and generate customized recommendations. More recently, artificial intelligence and generative AI have emerged as highly visible themes, reflecting the rapid adoption of advanced AI applications in digital marketing and online commerce. This suggests that researchers are now examining how intelligent systems can create personalized content, recommendations, and marketing messages that influence consumer decisions in real time.

At the same time, themes such as digital transformation, consumer, and customer experience continue to develop alongside the technological terms, showing that the literature is becoming more balanced between AI tools and consumer response. This is especially relevant for Millennials and Generation Z, who are highly active in online and mobile shopping environments and are frequently exposed to AI-based personalization strategies. The pattern indicates that the research field is moving from general consumer analytics toward more advanced, AI-driven, and context-sensitive studies of online behavior and impulsive purchasing.

The Scopus trend analysis shows a similar but more condensed development path. Artificial intelligence, consumer behavior, and electronic commerce are the most recent and frequently appearing themes, indicating that they currently dominate the field. Machine learning and online consumer behavior appear earlier and remain relevant over time, suggesting that they form the conceptual base of the literature. Themes such as personalized recommendation and buying behavior show moderate but growing attention, which points to emerging interest in the behavioral consequences of AI-powered personalization. Overall, the Scopus pattern confirms a shift from broader consumer studies toward more data-centric and AI-driven research in e-commerce settings.

**Figure 15:**
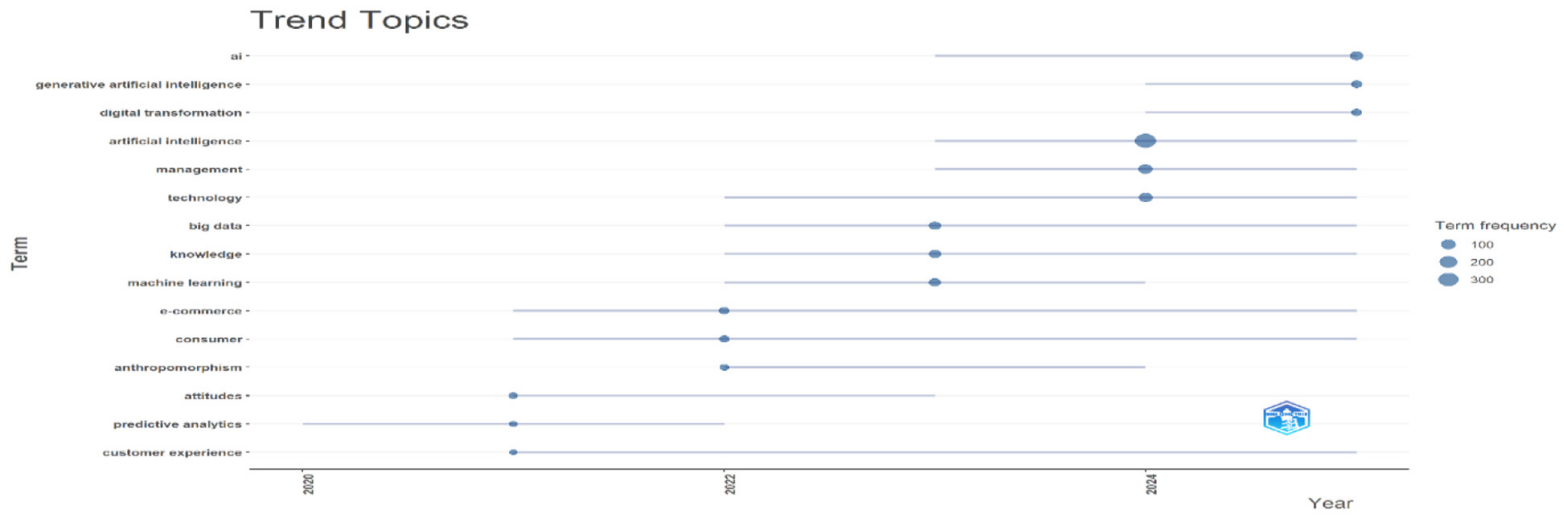
Trend topics.

Taken together, both datasets show the same broad movement: the field has evolved from predictive and consumer-experience-based studies toward more advanced AI, machine learning, and personalization themes. Web of Science highlights the newer appearance of generative AI and the continuing role of customer experience, while Scopus shows the stronger present-day dominance of artificial intelligence, consumer behavior, and electronic commerce. The combined insight is that AI-driven personalization and impulsive buying is becoming a more mature and technology-intensive research area, with growing attention to how digital systems shape consumer behavior in online markets (see Figure 15).

### 3.6 Thematic and intellectual structure

#### 3.6.1 Thematic map analysis

According to the Web of Science thematic map, it can be seen that the field is still at its conceptual stage and has not yet acquired a fully integrated core theme that relates AI technologies to impulsive buying behavior. The cluster of such themes as artificial intelligence, management, and technology falls in the niche themes quadrant, i.e., it is an internally developed, yet a fairly peripheral part of the overall research network. This implies that much of the literature is centered on AI in a technological or managerial context, without necessarily relating it to the end-user results. Conversely, the cluster of interest, which is trust-adoption-behavior is represented in the central themes quadrant as it shows that consumer response is still a significant and well-developed topic in the literature. This is particularly applicable to Millennials and generation Z, as the trust in algorithmic suggestions and the use of an AI-powered platform will probably affect the decision-making process online and potentially lead to impulse purchases. In general, the Web of Science map indicates that the discipline is still in its infancy, and there is a need to have a more solid connection between technological innovation and consumer behavior, particularly when it comes to research on digitally native generations.

The Scopus thematic map has a more advanced and strategically merged structure. The words that are most central and developed in the motor themes quadrant include: artificial intelligence, consumer behavior and electronic commerce. It means that there is already a solid intellectual core of the field, where AI and consumer behavior are tightly related, via e-commerce research. The fact that your topic lies in a dynamic and well-linked field is indicated by such terms as personalized recommendation, impulse buying and online consumer behavior, which are right next to your topic. Specialized areas of the niche themes quadrant are like financial services and services marketing, which are developed and not as closely related to the wider area. In the meantime, new or fading trends like explainable AI, impulsive buying behavior, and the control of the budget imply opportunities with which the future research may proceed. Themes like the use of social media, sustainability and consumption behavior are basic and developing, which implies that they will be used to support the field without constituting the core.

**Figure 16:**
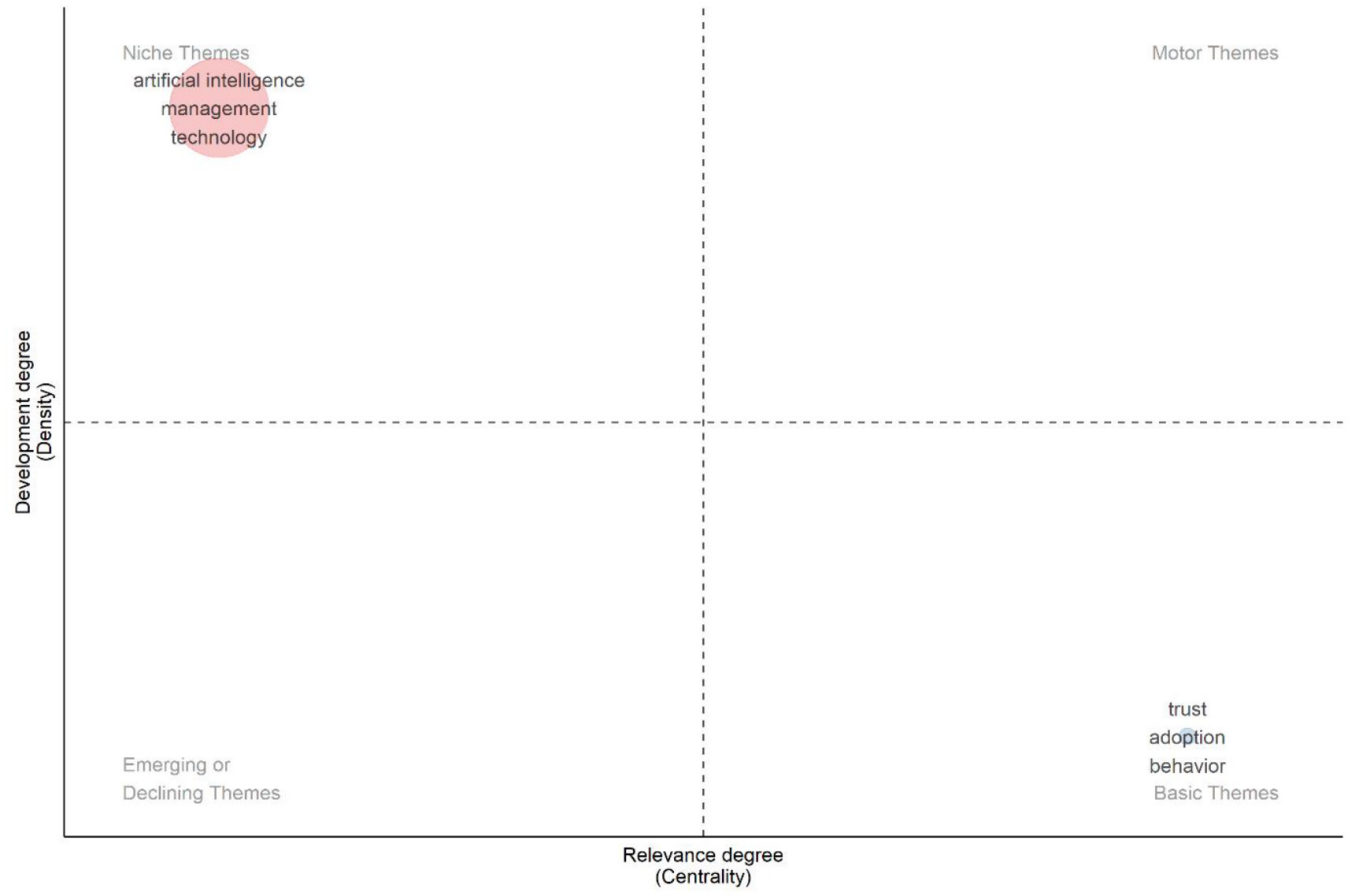
Thematic map analysis.

Combined, both of the datasets demonstrate that the field is gravitating toward closer relations between AI and consumer behavior, but they vary in terms of maturity. Web of Science indicates that the field is in need of a fully integrated core between technology and impulsive buying, whereas Scopus indicates a more developed thematic organization where AI, consumer behavior, and e-commerce are already the drivers of the field. The collective wisdom is that AI-based personalization and impulse buying is a developing interdisciplinary field with immense possibilities in the future, particularly when it comes to future efforts on Millennials and Generation Z (see Figure 16).

#### 3.6.2 Factorial analysis

The factorial analysis of the Web of Science, which is based on Multiple Correspondence Analysis, demonstrates that the intellectual framework of the field is constructed on the basis of two major conceptual orientations. On the one hand, the consumer-centered cluster encompasses the keywords e-commerce, consumers, trust, satisfaction, privacy, user acceptance and behavior. This cluster represents the studies that concentrate on the interactions of digital consumers with personalized online space and influence of psychological and behavioral factors on online buying behavior. This is particularly relevant to Millennials and generation Z who are very active in the digital marketplace and are highly subjected to AI-based personalization. The technology-oriented cluster comprises artificial intelligence, big data, machine learning, analytics, digital transformation, algorithms, and automation on the other side. These terms indicate that the technical background of individualized recommendation systems and data-driven marketing solutions are in the focus of much of the literature. On the whole, the Web of Science map indicates that the field has yet to experience a conceptual shift whereby the notion of technological innovation and consumer behavior are more loosely connected, with one stream oriented toward the development of AI-based systems and the other toward the perception of their impact on impulsive buying.

This interpretation is reinforced as the dendrogram reveals the keywords grouping them into hierarchical clusters that generally relate to the same two streams of research. It demonstrates the combination of certain issues into larger themes, in particular, technology-oriented research and consumer behavior-oriented research. This trend indicates that the area is evolving with the overlap of two mutually supporting viewpoints: artificial intelligence as the technological facilitator and consumer behavior as the result domain. This is especially applicable to the Millennials and generation Z individuals since they are used to interacting with algorithmically personalized online space, where trust, adoption and behavioral reaction can come into play and affect impulse buying. The factorial analysis thus indicates that literature is increasingly becoming interlinked, yet the dynamics between technological development and consumer response remains unassimilated.

The factorial map given by Scopus has a more closely knit conceptual framework. It depicts a big, highly intertwined cluster where artificial intelligence, consumer behavior, machine learning, impulse buying, and e-commerce are closely intertwined, meaning that the sector is already being focused on AI-based consumer analytics. Smaller sub-themes can be detected within this core. Such terms as machine learning, decision making, data mining and purchase behavior are a more technological and analytical subgroup whereas impulse buying, online consumers and social commerce are more behavioral and marketing oriented. Other terms like sustainability, perceived value, and social media seem to be somewhat detached indicating they are novice extensions of the overall research stream. The data privacy, trust and recommendation agent are more distanced on the periphery, which indicates that these issues are not well established within the mainstream research rhetoric yet. This structure is confirmed by the dendrogram which indicates how the field divides into a small number of major thematic groups and converges at higher levels. It implies three general streams AI and technological applications, consumer behavior and decision-making, and digital commerce environments.

**Figure 17:**
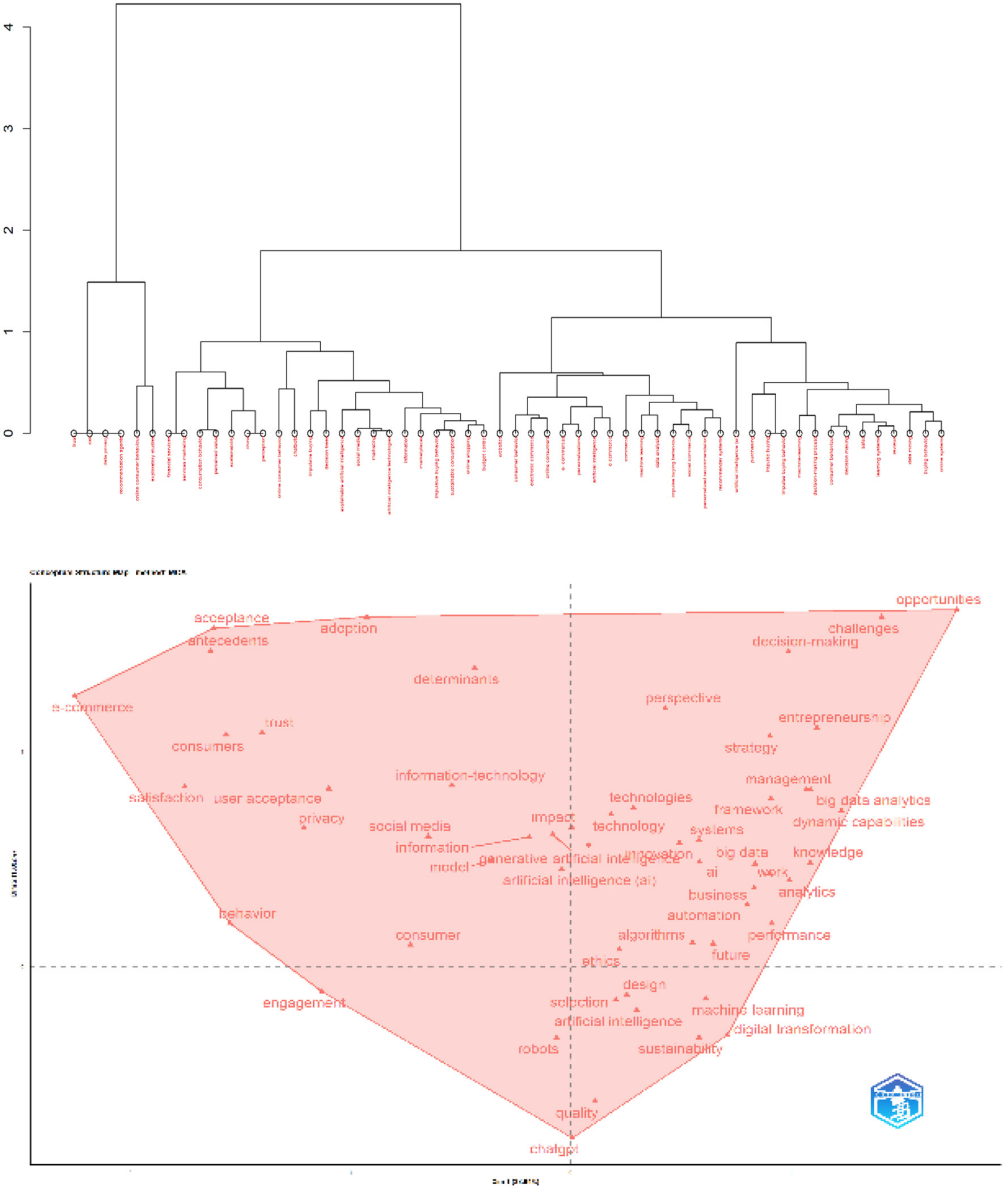
Factorial analysis from WOS data set analysis.

When combined, the two datasets reveal that the field is well-organized yet developing. Web of Science offers a better distinction between technology and behavior whereas Scopus offers a more coherent structure where the latter are already tightly intertwined. The synthesized understanding is that AI-based personalization and impulse purchase is a fast-growing interdisciplinary field, and conceptual connections between technological innovation, consumer reaction, and online trade are slowly shifting toward the center, as are new components like personal privacy, sustainability, and recommendation systems (see Figure 17).

## 4 Discussion

The intellectual framework and research focus of the personalization and impulsive purchasing behavior based on artificial intelligence was studied based on a bibliometric analysis of 650 articles in the Web of Science and 48 articles in Scopus, written between 2020 and 2025, and further validated by OpenAlex and Dimensions. The results indicate that the field of study is growing at a fast rate and gaining more academic interest in such fields like digital marketing, consumer behavior, information systems, and data analytics.

To begin with, the findings show that the AI-based personalization research boasts a significant upward trend. The gradual rise in the number of publications, as well as the trend toward increased growth, indicates that researchers are becoming more interested in the impact of artificial intelligence on consumer decision-making in the online setting (Amin, 2025). Such expansion is indicative of the increasing application of the algorithmic recommendation system and tailored marketing strategy within e-commerce platforms, especially the Millennials and Generation Z, who are the most active participants in the online shopping environment (Shalini, 2025).

Second, the field is multidisciplinary and international as indicated in the analysis. The comparatively good degree of international cooperation indicates that the scientists of various nations are working on the evolution of this field. This kind of cooperation is common in new interdisciplinary fields in which technological knowhow in artificial intelligence is combined with behavioral and marketing research (Fadilah et al., 2025). The wide writing audience also gives evidence of a high level of scholarly interest in learning about the behavioral effects of algorithmic personalization.

Third, the thematic and key word analysis indicates that there are a few common themes in research. The technological foundations of the field are reflected in such core topics like artificial intelligence, digital transformation, big data, and technology management (Cavazos-Arroyo and Máynez-Guaderrama, 2022). Meanwhile, more recent topics like recommender systems, predictive analytics, consumer experience, and impulsive buying behavior signify an increased interest in the impact of AI-oriented systems on consumer psychology and buying behavior in an online environment.

Lastly, the time trend analysis indicates that advanced AI applications, such as generative artificial intelligence and intelligent recommender systems, are becoming of greater concern in the recent studies. This transformation is indicative of the fast changing nature of digital commerce technology and the increased significance of customization in the development of consumer interaction and spontaneous buying behavior (Amin, 2025). Overall, the results indicate that the study of AI-based personalization and impulsive purchase is in a fairly young yet incredibly fast-evolving phase with an interdisciplinary expansion and shifting theoretical perspectives.

### 4.1 Theoretical implications

The results of this bibliometric analysis have multiple theoretical implications to consumer behavior, AI-driven marketing and research on digital retail. To start with, the research adds value to the body of knowledge on consumer behavior by revealing the increasing power of the AI-based personalization as a driver of consumer choice (Fale, 2025). Conventional theories in consumer behavior have been mostly concerned with human-based marketing cues, although the growing popularity of automated recommendation systems and predictive analytics indicates that AI-mediated decision contexts are now significant in influencing consumer decisions (Avinash et al., 2025). It requires the extension of the current theoretical models to encompass algorithmic persuasion, AI-mediated communication, and digitally organized choice environments (Effendi et al., 2025). Theories of future consumer behavior must thus consider the influence of AI-based suggestions on attention, preference formation, and impulse buying behavior on the Internet.

Second, the paper contributes to the theoretical advancement of AI marketing, demonstrating that artificial intelligence, big data, and digital transformation are taking center stages in the modern marketing practice. This tendency implies that the technological views should be incorporated more extensively into the classical marketing theory. The personalization systems based on AI are used by continuous learning processes driven by data, which adjust to the consumer behavior and, thus, redefine the conventional relationship between firms and consumers (Shyni et al., 2025). Due to this, the idea of algorithmic personalization, automated decision making, and data-driven engagement should be center-stage in marketing theory.

Third, the research has some implications to the theory of digital retail especially concerning online communication and online shopping behavior. The development of digital retail environments to affect consumer choices with the introduction of themes like predictive analytics, recommender systems, and customer experience suggests that these environments are becoming more and more consumer-focused in their design. This implies that the digital retail theory needs to transcend the traditional models of online shopping and integrate AI-powered retail mechanisms where algorithms take an active role in product discovery and purchase choices. In that respect, impulsive buying must be interpreted not just as an impulsive psychological reaction, but partially as a technologically mediated behavior, which is provoked by the recommendation system (Shalini, 2025).

### 4.2 Practical implications

The research has also significant practical implications to e-commerce platforms, marketing practitioners, and creators of AI-based recommendation systems. To begin with, the results are very applicable in e-commerce websites. The increased popularity of artificial intelligence, digital transformation, and consumer behavior indicate that the AI-driven personalization has become a vital aspect of online shopping strategy (Jancy and Muthukumaravel, 2025; Shalini, 2025). Machine learning and sophisticated analytics can help e-commerce companies learn more about the consumer preferences, browsing and purchase behavior, offering them a chance to provide more appropriate recommendations, optimize product discovery, and improve the overall customer experience (Ramesh and Murugan, 2025).

Nevertheless, the findings also indicate that excessive personalisation can promote unthought-out and unplanned buying. Over targeting can inhibit deliberation and increase the effect of cognitive biases, leading to distrust in case the consumer feels manipulated (Avinash et al., 2025). Thus, platforms must strike a balance between personalized recommendations and sustainable design methods that promote consumer agency and build a long-term loyalty (Yadav, 2025).

Second, the research has some implications to marketing practitioners. The growing use of AI in marketing implies that specialists need to become more data-driven in their decision-making. One-to-one communication, personalized marketing, and predictive campaigns may make the process of engagement and conversion rates better when applied successfully (Ghule, 2025). Meanwhile, the results demonstrate the significance of being transparent and ethically responsible, especially since consumers might also be worried about privacy, bias in the algorithm, and over targeting.

Third, the research can be used in designing and developing AI-based recommendation systems. The popularity of predictive analytics, big data, and recommender technologies demonstrates that these technologies are at the center of influencing consumer purchasing behavior within online settings (Gunawan, 2025). The developers should then aim at enhancing the accuracy, relevance and diversity of recommendations (Cui and Mohib, 2025). Moreover, explainable AI will enable the establishment of consumer trust as the process of recommendations will be more transparent (Effendi et al., 2025). These changes can make the recommendation systems more accountable, consumer-focused, and effective in helping with the digital retail approaches (Gupta, 2025).

### 4.3 Limitations

Even though the current research is a significant addition to the body of knowledge related to AI-based personalization and impulsive purchasing behavior, a few limitations must be recognized. To start with, the analysis will be performed using a specific collection of records retrieved using Web of Science and Scopus and validated using OpenAlex and Dimensions. Although these sources are a good coverage of high-quality literature, not all the relevant studies published in every database and repository are captured in these sources. Consequently, there is the possibility of the omission of certain relevant research (Digiampietri et al., 2025).

Second, only English language publications were sampled. This limitation might have omitted valuable research published in other languages and consequently not be representative of non-English speaking areas, even though English is the most used language of scholarly communication.

Third, the research was dedicated to 2020-2025. Although this period includes the recent advances in AI-based marketing and online shopping, it might not cover the previous background research that influenced the theoretical framework of the area. Moreover, since the field of artificial intelligence is constantly progressing, the research trends will be even more altered in the next few years (Chen and Lasi, 2025). These shortcomings do not undermine the overall worth of the study, but they do imply that the results are to be approached with care.

The limitations of the study could be overcome in future studies with the help of adding more databases, considering non-English publications, and increasing the time frame of the analysis to get a wider picture of the research environment.

### 4.4 Possible directions of future research

The current bibliometric analysis shows that there are several promising avenues in the future research of AI-driven personalization and impulsive buying behavior among Millennials and Generation Z. One, the future research should explore the direct behavioral mechanisms by which AI personalization can impact impulsive buying and not just by considering personalization as an overall digital marketing instrument. This would assist in clarifying the relationship whether AI cues mediate the relationship between AI cues and impulse buying decisions (Ahmed et al., 2026a).

Second, there is a need of more empirical research that is specifically oriented at Millennials and Generation Z as distinct groups of consumers. The existing literature tends to put younger consumers in the same bracket or to include them in larger groups, which restricts the ability to understand the differences between generations in their level of digital responsiveness, AI sensitivity, and impulse control. In future studies, the comparison of these cohorts to platforms that are AI-powered cues, e.g. e-commerce websites, social commerce, and mobile applications that shop by artificial intelligence can be done to identify whether their responses to AI-driven cues differ in any meaningful way (Ahmed et al., 2026a).

Third, future research should include contextual and psychological variables like the type of the product, the structure of the platform, privacy issues, perceived manipulation, and post-purchase feelings. According to recent studies, not all cues of personalization have the same effect, and a more subtle approach should be used to elucidate when and how AI-based recommendations trigger impulsive purchasing (Anwar et al., 2024). Lastly, it would be worthwhile to conduct cross-cultural and cross-country studies due to uneven coverage of the geographical area and strong concentration of the few countries. Further research into underrepresented areas would enhance the applicability of the findings and determine the influence of the cultural and infrastructural differences on the AI-enabling consumer behavior (Hussain et al., 2025).

## 5 Conclusion

This paper presents a critical bibliometric evaluation of the literature on the topic of the personalization of impulse buying behavior using AI and the focus on Millennials and Generation Z. The study maps the intellectual structure, thematic development, and collaboration patterns of the field using 650 publications retrieved in Web of Science and 48 publications retrieved in Scopus and analyzed with the help of Biblioshiny and validated against other sources. The findings indicate that the study of artificial intelligence and online consumer behavior has increased significantly over the recent years, yet the particular connection between AI-driven personalization and the impulsive purchasing behavior has not been developed conceptually to its full extent (Cavazos-Arroyo and Máynez-Guaderrama, 2022). By systematically mapping the intellectual structure of this emerging field, the present study clarifies how technology-oriented and consumer-oriented streams are beginning to converge and identifies the missing conceptual links between algorithmic personalization and impulsive buying among digitally native consumers.

The comparison of the two datasets reveals that two streams, which are broad but interrelated, influence the field. The literature in the Web of Science data is more distinctly categorized into technology-oriented, including artificial intelligence, big data, machine learning, and digital transformation, and consumer-oriented, including trust, satisfaction, privacy, user acceptance, and behavior (Yadav, 2025). These themes seem to be more closely interrelated in the Scopus data, with artificial intelligence, consumer behavior, machine learning, impulse buying, and e-commerce having a more coherent conceptual core. Combined, these results indicate that the discipline is transforming into a much more integrated research discipline than it was formerly, which was a more parallel development of both technology and behavior research (Hussain et al., 2025). This bibliometric perspective thus advances the theoretical discussion by positioning AI-driven personalization not simply as a technical tool, but as a behavioral stimulus that should be explicitly integrated into models of online impulse buying for Millennials and Generation Z.

With this advancement, however, there is still a research gap. How AI-based personalization can have a direct impact on impulsive buying behavior in digitally native consumers remains unexplained in the literature (Chen and Lasi, 2025). Even though the adoption of technology and consumer trust are well accounted, the theoretical relationship between algorithmic personalization and spontaneous buying behavior should be addressed more. This is a critical point of connection since Millennials and Generation Z engage in online trading extensively, but are not usually addressed as their own cohorts (Cui and Mohib, 2025). Longitudinal and experimental designs, cross-cultural comparisons, and mixed-method studies could help move the field beyond descriptive correlations and toward causal and context-sensitive explanations of AI-induced impulse buying (Rai et al., 2025).

From a managerial perspective, the findings suggest that firms designing AI-driven personalization systems for Millennials and Generation Z should balance commercial objectives with consumer well-being, recognizing that finely tuned recommendations can both enhance convenience and intensify impulse buying (Ahmed et al., 2026a). Platform designers and marketers can draw on the emerging thematic clusters identified in this study to develop more transparent, controllable, and responsible personalization strategies, for example by incorporating choice architectures that support informed decisions rather than merely maximizing short-term impulsive purchases. For regulators and policymakers, the mapped research gaps highlight the importance of clearer guidelines around data usage, algorithmic transparency, and the protection of younger consumers in highly personalized digital environments (Ahmed et al., 2026a,b).

There are also certain limitations to the research. The study is limited by the reliance on articles indexed in Web of Science and Scopus within a defined timeframe and by the specific inclusion criteria applied. Future bibliometric and systematic reviews could extend this work by incorporating additional databases, gray literature, and non-English publications, and by updating the analysis as the field matures. All in all, the results point to the necessity to adopt more integrated and interdisciplinary strategies that can be used to bridge the gap between artificial intelligence technologies and the consumer behavior theory to create a more comprehensive picture of impulsive buying in the context of AI-driven personalization (Pal, 2025).

## Data Availability

The original contributions presented in the study are included in the article/supplementary material, further inquiries can be directed to the corresponding author.
